# Impact of a selective cyclooxygenase-2 inhibitor, celecoxib, on cortical excitability and electrophysiological properties of the brain in healthy volunteers: A randomized, double-blind, placebo-controlled study

**DOI:** 10.1371/journal.pone.0212689

**Published:** 2019-02-22

**Authors:** Jung-Ah Lim, Ki-Young Jung, Boram Park, Tae-Joon Kim, Jin-Sun Jun, Keun Tae Kim, Tae-Won Yang, Soon-Tae Lee, Keun-Hwa Jung, Kon Chu, Sang Kun Lee, Kyung-Il Park

**Affiliations:** 1 Department of Neurology, Comprehensive Epilepsy Center, Laboratory for Neurotherapeutics, Biomedical Research Institute, Seoul National University Hospital, Seoul, South Korea; 2 Department of Neurology, Gangnam Sacred Heart Hospital, Hallym University College of Medicine, Seoul, South Korea; 3 Department of Public Health Science, Seoul National University, Seoul, Korea; 4 Department of Neurology, Ajou University School of Medicine, Suwon, South Korea; 5 Department of Neurology, School of Medicine, Kyungpook National University, Kyungpook National University Chilgok Hospital, Daegu, South Korea; 6 Department of Neurology, Keimyung University Dongsan Medical Center, Daegu, South Korea; 7 Department of Neurology, Gyeongsang National University Changwon Hospital, Gyeongsang National University School of Medicine, Changwon, South Korea; 8 Department of Neurology, Seoul National University Hospital Healthcare System Gangnam Center, Seoul, South Korea; University of Toronto, CANADA

## Abstract

The inflammatory response is considered a defence mechanism against physical or infectious insults and is prevalent within the central nervous system. Seizures also result in a robust inflammatory cascade, leading to enhanced activation of excitatory synaptic networks. Ample evidence based on animal models of epilepsy has demonstrated that celecoxib, a highly selective inhibitor of cyclooxygenase-2, has anticonvulsant effects. We aimed to evaluate the impact of celecoxib on the cortical excitability and electrophysiological properties of the brain in healthy humans. Electroencephalography (EEG) or transmagnetic stimulation (TMS) was used to measure neurophysiological activity. Forty healthy volunteers were randomized to 4 groups (n = 10 in each group): 1) celecoxib and EEG, 2) placebo and EEG, 3) celecoxib and TMS, and 4) placebo and TMS. For the EEG study, resting EEG was performed at baseline just before administering 400 mg of celecoxib or placebo and repeated 4 hours after administration. The subjects took 200 mg of celecoxib or placebo twice a day for 7 subsequent days, and a third EEG was conducted 4 hours after the final dose. Power spectra were compared at each time point. For the TMS study, the resting motor threshold (RMT), motor evoked potential (MEP) peak-to-peak amplitude, and cortical silent period (CSP) were measured at baseline and after taking 200 mg of celecoxib or placebo twice a day for 7 days. Celecoxib did not significantly change brain activity in the EEG study. However, the sum of power recorded from all electrodes tended to increase in the celecoxib group only at 4 hours after administration (*p* = 0.06). In detail, one dose of celecoxib (400 mg) transiently and significantly increased the alpha band power recorded in the frontal and parietal areas as well as in the whole brain (*p* = 0.049, 0.017, and 0.014, respectively) and the beta frequency in the central and parietal regions (*p =* 0.013 and 0.005, respectively), whereas the placebo did not. This effect was abolished after 7 days of treatment. In the TMS study, we found no statistically significant change in the RMT, MEP peak-to-peak amplitude or CSP. This evidence suggests that celecoxib transiently alters the electrophysiological properties of the brain but does not suppress neuronal excitability in healthy humans.

## Introduction

Celecoxib is a highly selective inhibitor of cyclooxygenase-2 (COX-2) and a widely prescribed anti-inflammatory drug to alleviate the pain related to degenerative or rheumatoid arthritis. In addition to approved usage relating to its anti-inflammatory mechanisms of action, celecoxib has also been studied for repurposing as an anticancer drug [1] as well as for the treatment of epilepsy. Within the central nervous system, the inflammatory response is considered a defence mechanism against physical or infectious insults [2]. In addition to brain injuries such as trauma, ischemia, or hypoxia, seizures result in a robust inflammatory cascade leading to enhanced activation of excitatory synaptic networks [3]. Activation of glia and neurons by seizures rapidly induces a variety of pro-inflammatory cytokines, including cyclooxygenase-2 (COX-2) [3].

Celecoxib has been studied in several *in vivo* epilepsy models, including acute seizures [4–6], spontaneous recurrent seizures (SRS) [6], and kindling models [7]. Most studies have indicated anticonvulsant or neuroprotective effects of celecoxib [8–10], although a few studies have reported conflicting results [11, 12]. Celecoxib may have a suppressive effect on neuronal excitability as some inflammatory cytokines activate neuronal firing and promote neuronal excitability. One recent study demonstrated more direct evidence, where bath application of celecoxib on epileptic hippocampal slices suppressed neuronal activity and seizure frequency [13]. However, no study has been conducted on the electrophysiological effect of celecoxib in humans. Spectral analysis of electroencephalography (EEG) is a proven electrophysiologic tool to assess the pharmacological effects of drugs on the central nervous system, such as antiepileptic drugs [14, 15]. Additionally, repetitive transcranial magnetic stimulation (TMS) also provides information on changes in cortical function and increases in the motor evoked potential (MEP) amplitude and cortical silent period (CSP) by activating intracortical excitatory and inhibitory interneurons [16–18], respectively.

Utilizing EEG and TMS, we aimed to evaluate the impact of a single dose of and repetitive treatment with celecoxib on cortical excitability and the electrophysiological properties of the human brain in healthy volunteers.

## Materials and methods

### Participants

Forty healthy adults (5 females, 14 males, age 33.9 ± 7.3 years for the EEG study; 13 females, 7 males, age 32.9 ± 7.7 years for the TMS study) participated in this study. Participants were excluded if they met any of the following criteria: a) a history of seizure, cardiovascular disease (heart disease, stroke), hepatic disease, inflammatory bowel disease, or gastrointestinal hemorrhage; b) a body mass index (BMI) below 16.0 kg/m^2^ or greater than 30.0 kg/m^2^; c) a history of hypersensitivity to any medication; d) a history of any kind of medication within 1 week before screening; e) the presence of a clinically significant electrocardiogram abnormality at screening; f) an aspartate aminotransferase (AST) or alanine aminotransferase (ALT) level greater than 2.0× the upper normal limit; g) serum creatinine levels greater than 1.5× the upper normal limit; h) platelet counts lower than 100,000/μL; i) females who were pregnant, breastfeeding, or intended to become pregnant; j) a history of alcohol abuse; and k) participation in another drug study within 30 days before screening. The institutional review board of the Seoul National University Hospital approved this study, and written informed consent was obtained from the participants. The trial was registered in an international clinical trials registry, “ClinicalTrials.gov” (NCT02711579).

### Study design

**Fig 1:**
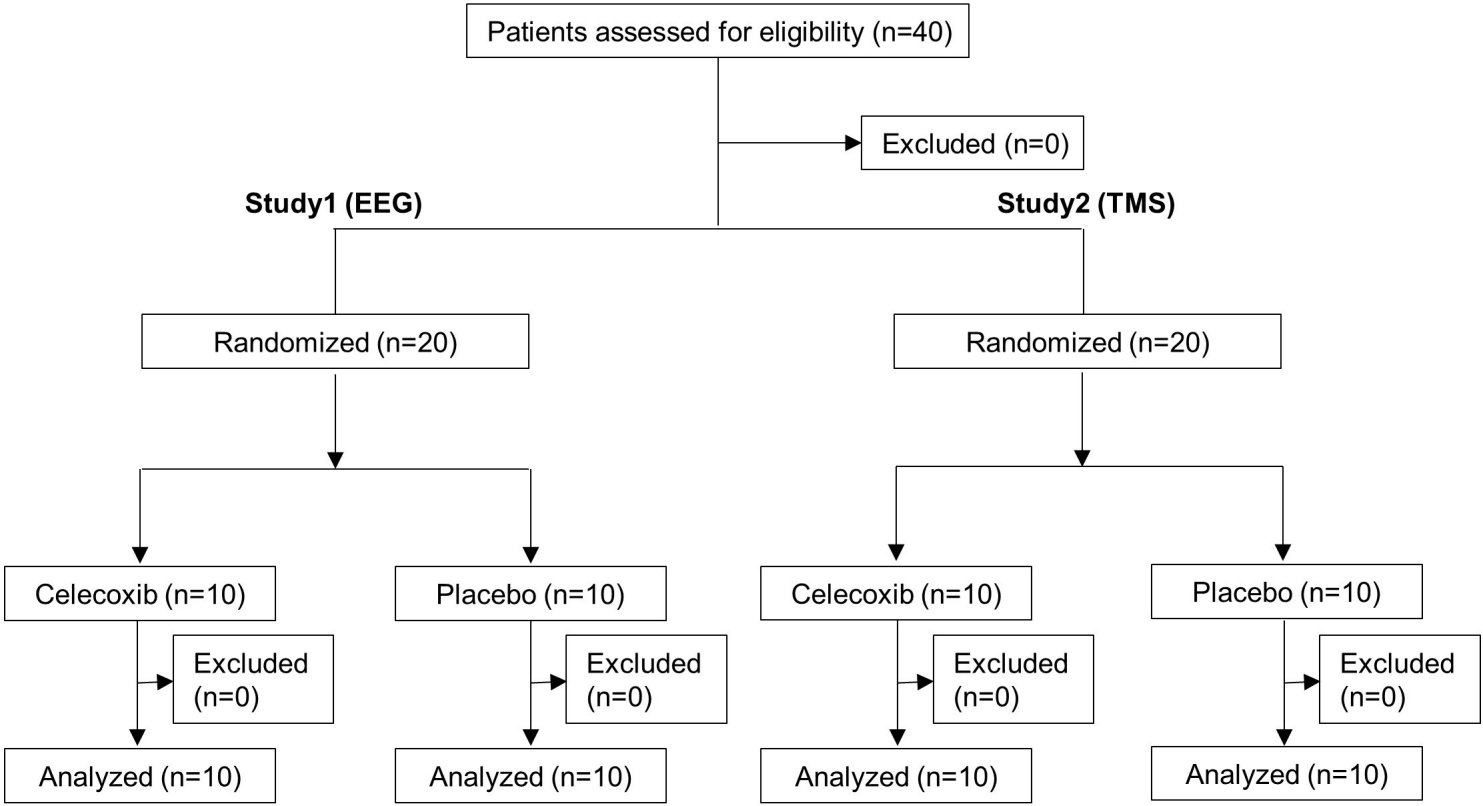
Study flow diagram. Abbreviations: EEG = electroencephalography, TMS = transmagnetic stimulation.

This was a randomized, double-blind, placebo-controlled study evaluating the impact of a single dose of and repetitive treatment with celecoxib on cortical excitability and the electrophysiologic properties of the brain in healthy volunteers and consists of two independent sub-studies. Each sub-study enrolled 20 subjects who were randomly assigned in a 1:1 ratio to receive celecoxib or placebo (Fig 1).

**Fig 2:**
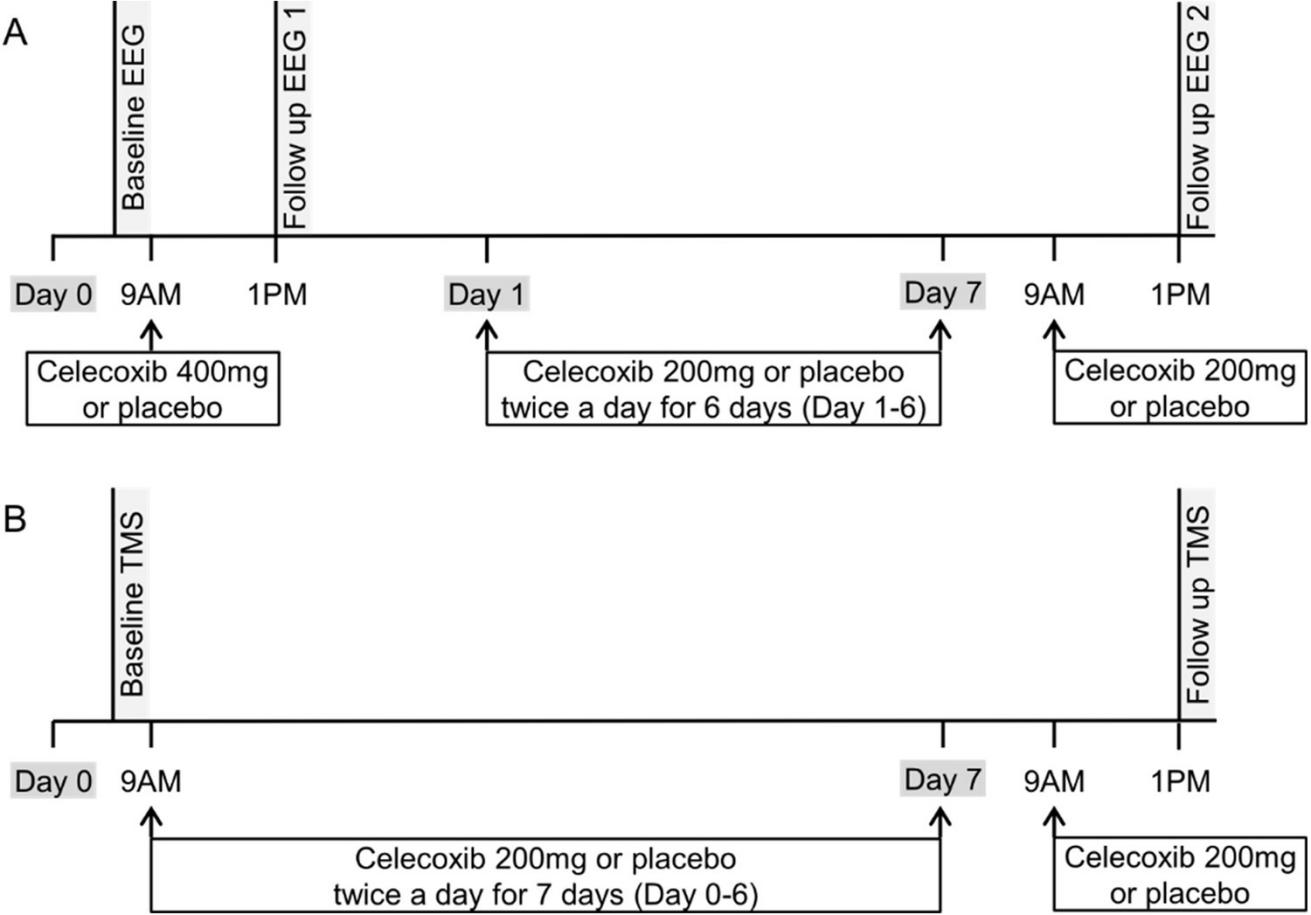
Schematic illustration of the study design. (A) Study 1 for electroencephalography. (B) Study 2 for transmagnetic stimulation. Abbreviations: EEG = electroencephalography, TMS = transmagnetic stimulation.

In study 1, EEG was performed at baseline just before celecoxib (400 mg) or placebo delivery at 9 AM, and then EEG was repeated at 1 PM, 4 hours after initial administration, to evaluate the acute electrophysiological response to a single dose of celecoxib. To evaluate the long-term effects of celecoxib, the subjects took celecoxib or placebo 200 mg twice a day for 7 subsequent days, and a third EEG examination was conducted at 1 PM on the last day, 4 hours after the final dose (Fig 2A).

In study 2, TMS was performed at baseline just before celecoxib or placebo (200 mg) delivery. The subjects took 200 mg of celecoxib or placebo twice a day for 7 days. The second TMS recording was performed 4 hours after the final dose (Fig 2B).

### Electroencephalography recording

EEG was recorded using a digital EEG system (Grass Technologies, Quincy, MA, USA) with a cap electrode placed according to the international 10–20 system (16 electrodes; Fp1, Fp2, F7, F3, F4, F8, T7, C3, C4, T8, P7, P3, P4, P8, O1, and O2). A two-channel electrooculogram (EOG) was placed on the left and right outer canthi to confirm eye movements and remove EOG artefacts. A linked-ear electrode was used as a reference. The impedances of all electrodes were kept below 10 kΩ. Electrical activities were sampled at 400 Hz, with bandpass filtering at 0.5–70 Hz. Resting-state EEG was recorded for 10 minutes while all patients were awake and alternately opened and closed their eyes every 1 minute [19].

### Electroencephalography power spectral analysis

The waking EEGs from each subject were analysed after termination of the entire study. EEG data were processed using EEGLAB version 10.0b in MATLAB (MathWorks, Natick, MA, USA). The EEG data were bandpass-filtered (0.5–50 Hz) after re-referencing to the common average reference for analysis, and 10 epochs of 1.5 seconds in duration in the eyes-closed state without artefacts were selected. For power spectral analysis, a fast Fourier transformation was performed using Welch’s method (Hamming window, 50% overlap). The band widths of delta, theta, alpha, beta, and gamma were 0.5–4.0, 4.0–8.0, 8.0–13.0, 13.0–30.0, and 30.0–50.0 Hz, respectively. Electrodes were grouped into frontal (F3 and F4), central (C3 and C4) and parietal (P3 and P4) regions. Changes in brain activity by celecoxib in the early (just after a single dose of celecoxib) and late (after a 7-day course of celecoxib) periods were assessed according to the brain location and band frequency.

### Transcranial magnetic stimulation

The MEP was measured using surface electromyography (EMG) electrodes placed over the first dorsal interosseous (FDI) muscle. A figure-of-eight shaped magnetic stimulator (Magstim 200 device, Magstim Co. Ltd., Dyfed, S. Wales, UK) was located over the contralateral motor cortex to produce optimal motor potentials in the FDI muscle. The resting motor threshold (RMT) was the lowest intensity required to evoke MEPs in the FDI muscle with a peak-to-peak amplitude of at least 50 μV in 4 of 8 trials [20]. With the FDI muscle in the relaxed state, stimuli were delivered to evoke MEPs at stimulus intensities of 120%, 140%, and 150% (if possible) of the RMT [21]. Eight stimuli were delivered randomly at each stimulus intensity every 5 seconds, and the peak-to-peak amplitudes were measured. To measure the CSP, the subjects were asked to produce 30% of their maximum voluntary contractions. While the subjects were contracting their FDI muscle, TMS stimuli at intensities of 120%, 140%, and 150% (if possible) of the RMT were delivered randomly 5 seconds apart, with 8 stimuli at each stimulus intensity. The CSP duration was defined as the time between the end of the stimulus-induced MEP and the reoccurrence of voluntary EMG activity.

### Study outcome

The primary outcome was a change in electroencephalography power spectra due to a single oral dose of celecoxib (400 mg) for study 1 (EEG), and a change in cortical excitability variables due to long-term treatment with celecoxib (200 mg twice daily for 7 days) for study 2 (RMS). The secondary outcome was a change in electroencephalography power spectra due to long-term (7 days) treatment with celecoxib for study 1 (EEG).

### Statistical analysis

Subject information and TMS data are described as the mean and standard deviation, while EEG power is described as the median and interquartile range (IQR). The Wilcoxon signed rank test and a linear mixed-effects model were used to evaluate the effects of celecoxib on EEG and TMS parameters. Repeated measures ANOVA was used to compare the frequency band and location for EEG data and different stimulus intensities for TMS parameters. Multiple comparisons were corrected by the Bonferroni method. Two-tailed *p* values < 0.05 were considered statistically significant. All statistical comparisons were performed with SPSS version 18.0 (SPSS Inc., Chicaco, IL, USA).

## Results

### Electroencephalography power spectra

**Fig 3:**
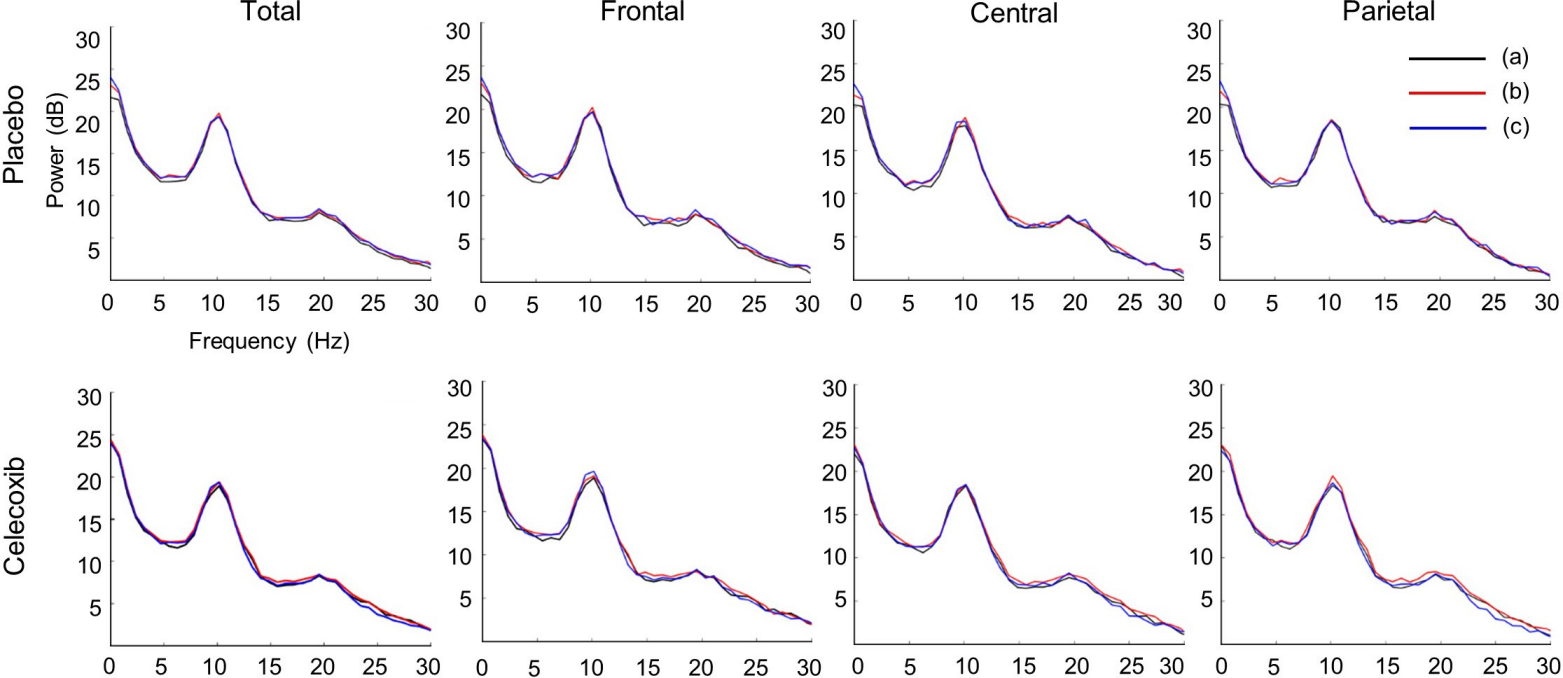
Averaged power spectra of the subjects receiving placebo (upper) or celecoxib (lower) at different recording sites. (a) Baseline, (b) after a single dose of placebo or celecoxib (400 mg), (c) after a 7-day treatment regimen.

**Fig 4:**
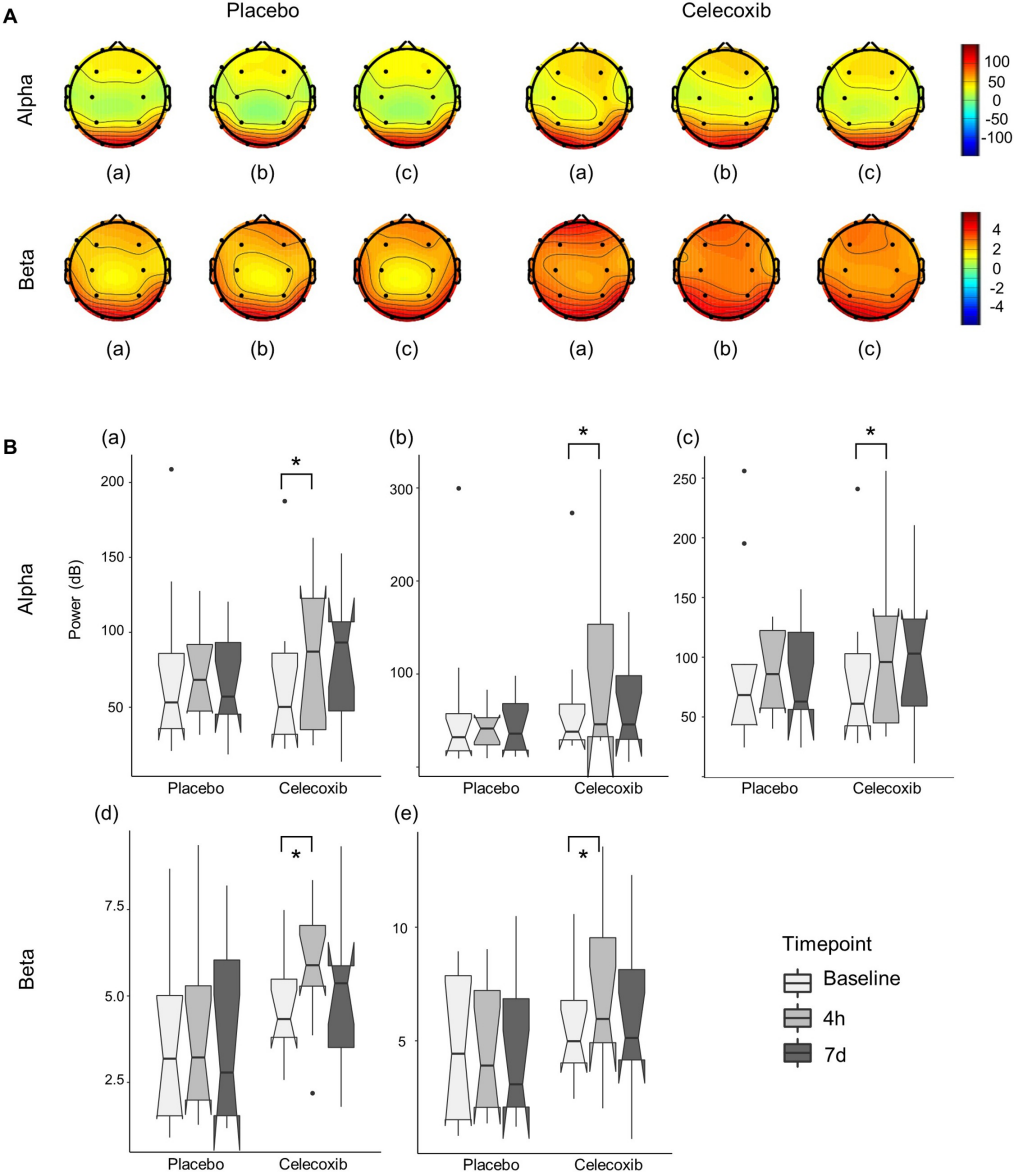
Power changes in the alpha and beta bands by celecoxib treatment. (A) The topography of EEG spectral power differences in alpha (upper) and beta (lower) frequency bands at baseline (a), after a single dose of placebo or celecoxib (400 mg) (b), and after a 7-day treatment regimen (c). (B) Celecoxib (400 mg once) treatment increased the power of the frontal lobe (a), parietal lobe (b), and total brain (c) areas at the alpha band and beta frequency in the central (d) and parietal (e) regions. However, this effect was not evident after 7 days of treatment (200 mg twice a day). Asterisks denote statistical significance (Wilcoxon signed rank test, *p* < 0.05). Abbreviations: 4 h, 4 hours; 7 d, 7 days.

All participants completed the study, and no adverse effects were reported. After administration of a single dose (400 mg) of celecoxib, no significant difference was found between the groups in the baseline power analysis in any of the brain regions at any of the frequency bands (Table 1 and Fig 3). The sum of power recorded from all electrodes was not altered significantly by celecoxib treatment, while it showed an increasing trend in the celecoxib group, especially at 4 hours after initial administration (*p* = 0.06). When analysed by frequency band and location, a single dose of celecoxib (400 mg) significantly increased alpha activity in the frontal lobe (50.24 [IQR 29.72–93.15] dB → 87.12 [IQR 31.12–134.81] dB, *p* = 0.049) and parietal lobe (38.03 [IQR 27.37–77.49] dB → 45.95 [IQR 32.05–160.73] dB, *p* = 0.017) as well as the whole brain (61.08 [IQR 40.09–111.60] dB → 91.00 [IQR 39.70–153.59] dB, *p* = 0.014), whereas the placebo did not (Fig 4). Additionally, one dose of celecoxib transiently increased beta activity in the central lobe (4.33 [IQR 3.52–5.67] dB → 5.88 [IQR 4.93–7.35] dB, *p* = 0.013) and parietal lobe (4.98 [IQR 3.76–7.47] → 5.96 [IQR 4.70–10.10], *p* = 0.005); however, this effect was transient and abolished after 7 days of treatment.

**Table 1:** Total electroencephalography power spectral density (absolute value).

	celecoxib			placebo		
	median	interquartile range	*p* value	median	interquartile range	*p* value
baseline	210.61	139.04–255.92		152.36	113.01–263.59	
400 mg once	257.42	184.93–391.88	0.06	201.66	159.76–241.81	0.11
7-day treatment	203.22	153.23–395.08	0.39	182.58	145.28–305.85	0.24

*p*-values represent the results compared with baseline by the Wilcoxon signed rank test

### Transcranial magnetic stimulation parameters

**Fig 5:**
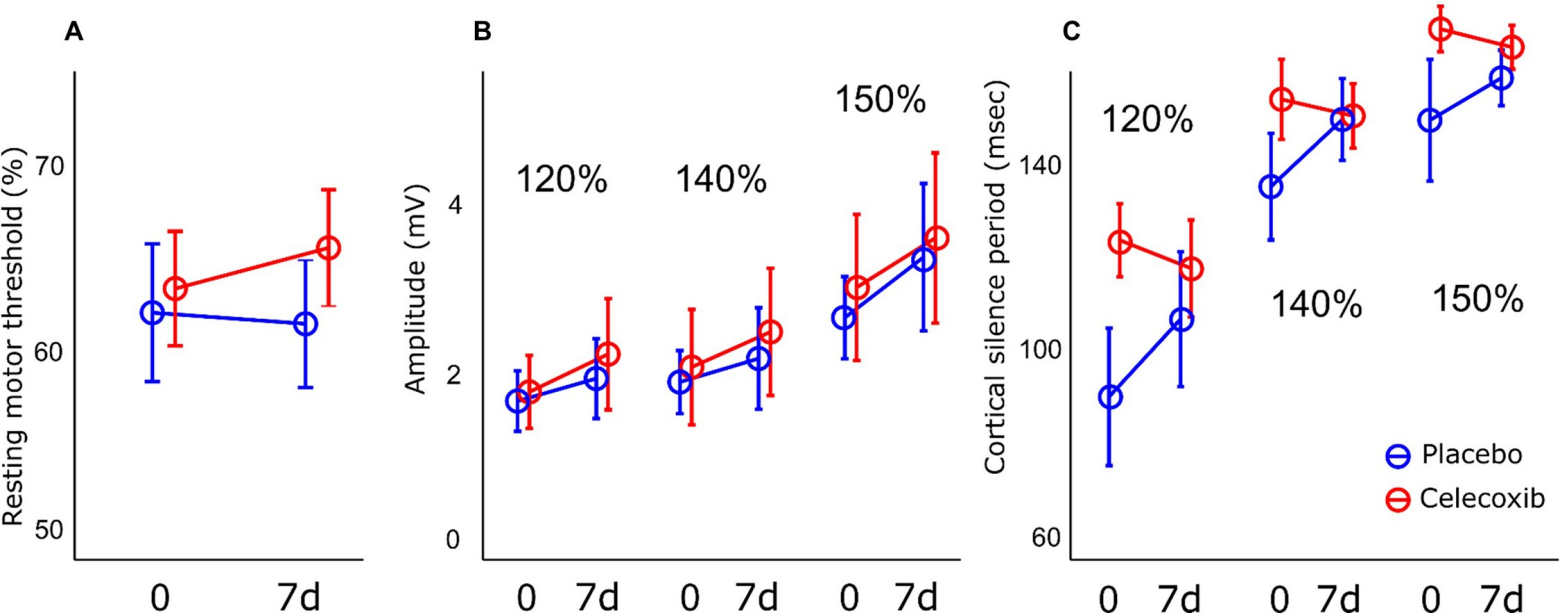
Transmagnetic stimulation parameters. No significant effects of 7-day treatment with celecoxib were found on the (A) resting motor threshold, (B) peak-to-peak amplitude, or (C) cortical silence period.

No statistically significant change was observed in any cortical excitability variables; the RMT (placebo 61.5 ± 10.9% vs celecoxib 65.6 ± 9.9%, *p* = 0.315), peak-to-peak amplitude of 120% (placebo 1.92 ± 1.53 mV vs celecoxib 2.22 ± 2.1, *p* = 1.000), peak-to-peak amplitude of 140% (placebo 2.62 ± 1.90 mV vs celecoxib 2.94 ± 2.39 mV, *p* = 0.912), peak-to-peak amplitude of 150% (placebo 3.83 ± 2.47 mV vs celecoxib 4.05 ± 2.48 mV, *p* = 0.662), CSP of 120% (placebo 107.0 ± 46.0 msec vs celecoxib 118.0 ± 33.1 msec, *p* = 0.684), CSP of 140% (placebo 140.5 ± 28.0 msec vs celecoxib 141.3 ± 22.0 msec, *p* = 1.000), or CSP of 150% (placebo 152.8 ± 19.5 msec vs celecoxib 160.4 ± 13.4 msec, *p* = 0.491). The overall distribution patterns of the data are displayed in Fig 5. Neither celecoxib nor placebo treatment had an effect on the MEP amplitude during repetitive TMS (*F* = 0.75, *p* = 0.540 for 120% of the RMT; *F* = 0.44, *p* = 0.709 for 140% of the RMT; *F* = 1.09, *p* = 0.367 for 150% of the RMT).

## Discussion

This study evaluated the effects of celecoxib on cortical excitability in the brains of healthy adults. A single dose of celecoxib transiently increased the alpha power of the frontal lobe, parietal lobe, and total brain areas and the beta power of the central and parietal areas; however, the effect did not last long.

The effect of celecoxib on neurophysiological activity can be inferred from animal epilepsy models, but previous studies have reported inconsistent results [8–13] depending on the epilepsy models used and the treatment protocols employed. Celecoxib reduced the likelihood of SRS when administered in the latent period in a pilocarpine-induced chronic epilepsy model [9]. In another chronic epilepsy model induced by kainate, administration of celecoxib during the chronic phase caused a significant reduction in SRS [13]. In pharmaco-resistant epilepsy, pre-treatment with celecoxib restored pharmaco-sensitivity [10]. In contrast, some other studies showed that COX-2 inhibition did not affect acute seizures but had a neuroprotective effect with post-treatment after kainate administration (7). Even worse, celecoxib exaggerated kainate-induced acute seizures with pre-treatment before kainate [11]. However, a recent study demonstrated more direct evidence using an *in vitro* model of epilepsy that celecoxib inhibited neuronal excitability, thus showing an anti-seizure effect [13]. As some inflammatory cytokines, such as prostaglandin (PG) E2 and interleukin-1β (IL-1β), are known to promote neuronal firing and exacerbate neuronal excitability [3], celecoxib may have anticonvulsive effects targeting inflammatory mediators. Based on most previous results, we hypothesized that celecoxib may have a suppression effect on neuronal excitability in humans. However, celecoxib did not suppress neuronal excitability contrary to our expectation.

Instead, celecoxib transiently increased the alpha power in this study. Alpha band waves on resting EEG play a role in maintaining an alert state [22]. Therefore, the increase in alpha power may be attributed to diurnal alteration of vigilance and does not reflect an effect of celecoxib. However, our exam times were homogenous among subjects, and the placebo group did not demonstrate such patterns, which may imply a potential effect of celecoxib on alertness. Some inflammatory cytokines are known to be involved in wake-sleep regulation [23, 24]. IL-1β and PGD2 are endogenous sleep factors, and selective COX-2 inhibition blocks the sleep-promoting effect of IL-β [25, 26]. The influence of cytokines on wakefulness regulation may result in increased alpha power mediated by celecoxib in our study.

This study is the first to evaluate the electrophysiological effect of COX-2 inhibition on healthy human brains. Although the study showed negative results, the data are meaningful because previous *in vitro* and *in vivo* studies reported contradictory results that led to a reluctance to use celecoxib for the treatment of human epilepsy.

Nevertheless, this study has a few limitations. First, the study population was relatively small. However, this study has strength because it was designed as a randomized, double-blind, placebo-controlled investigation. Second, whether any anticonvulsant effect of celecoxib on the epileptic human brain exists with increased inflammation remains unknown as we evaluated healthy volunteers. Third, we confirmed the first drug dose by in-person administration between the 1^st^ and 2^nd^ EEG examinations, but we did not verify drug compliance for 7 days using an objective method, such as blood level testing. Any non-compliance by subjects may affect the long-term results of EEG and TMS.

In conclusion, selective COX-2 inhibition by celecoxib transiently alters the electrophysiological properties of the brain but does not suppress neuronal excitability in healthy humans. Despite many animal studies reporting the anticonvulsive effect of selective COX-2 inhibitors, further investigation is required to demonstrate their effects in the treatment of human epilepsy.

## Supporting information

S1 File CONSORT 2010 checklist_CXB. (DOC)

S2 File CONSORT 2010 flow diagram_CXB. (DOC)

S3 File Study protocol_Engligh. (DOCX)

S4 File Study protocol_original language. (DOCX)
