# Supplementary figures and images for: Impact of a selective cyclooxygenase-2 inhibitor, celecoxib, on cortical excitability and electrophysiological properties of the brain in healthy volunteers: A randomized, double-blind, placebo-controlled study
Source: PLoS One. 2019 Feb 22;14(2):e0212689. doi: 10.1371/journal.pone.0212689 (PMC6386435; doi:10.1371/journal.pone.0212689)

**
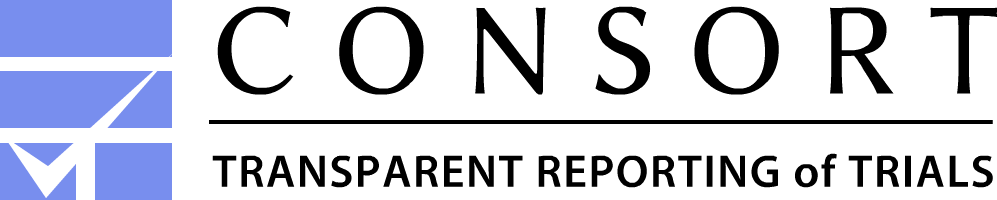
**


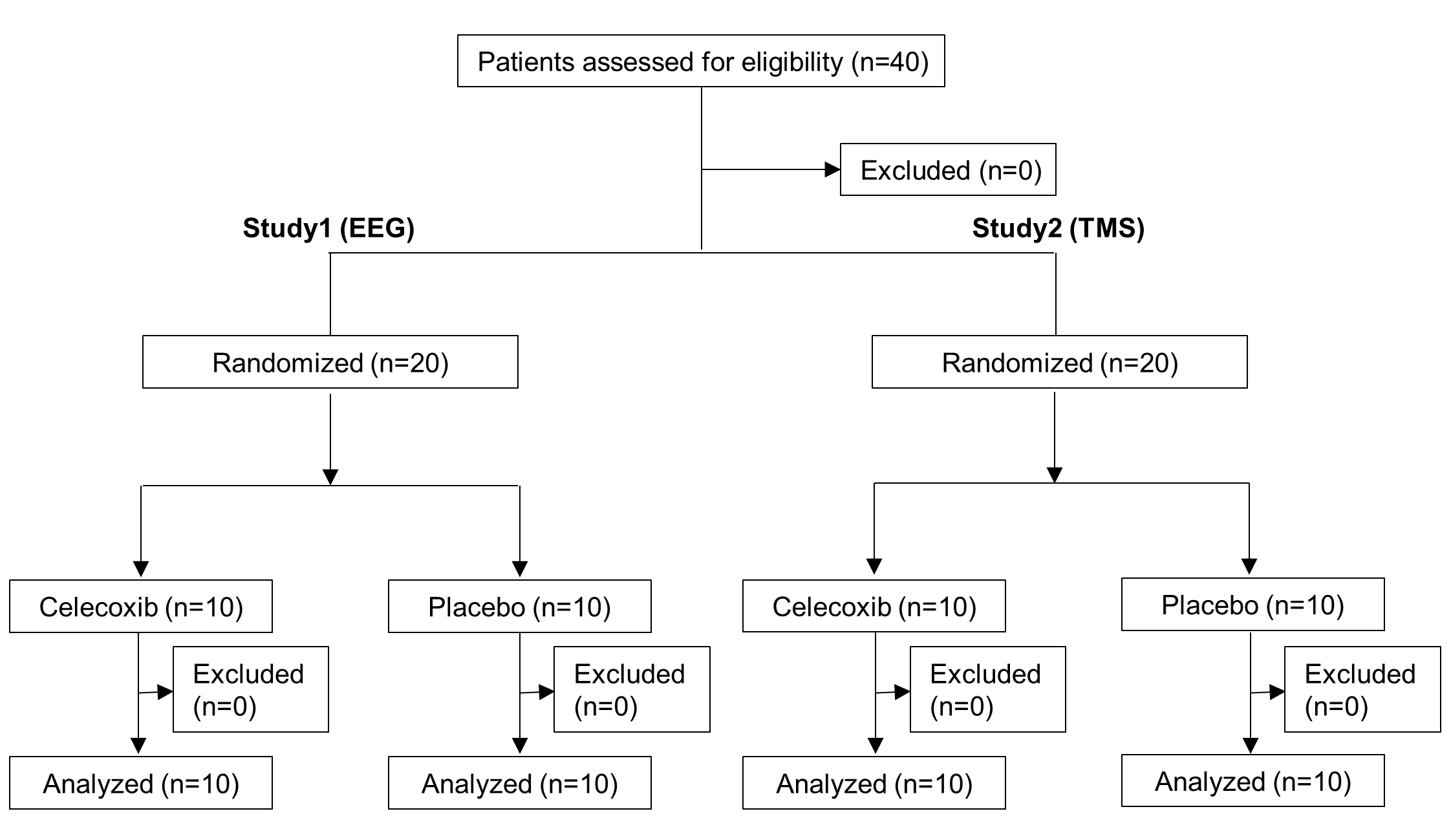
**CONSORT 2010 Flow Diagram**

**Allocation**

**Analysis**

**Follow-Up**

**Enrollment**

Supplement: S2 File — (DOC) [file pone.0212689.s002.doc]
