# Supplementary material for: Impact of a selective cyclooxygenase-2 inhibitor, celecoxib, on cortical excitability and electrophysiological properties of the brain in healthy volunteers: A randomized, double-blind, placebo-controlled study
Source: PLoS One. 2019 Feb 22;14(2):e0212689. doi: 10.1371/journal.pone.0212689 (PMC6386435; doi:10.1371/journal.pone.0212689)
Supplement: S3 File — (DOCX) [file pone.0212689.s003.docx]

**Impact of a selective cyclooxygenase-2 inhibitor, celecoxib, on cortical excitability and electrophysiological properties of the brain in healthy volunteers: A randomized, double-blind, placebo-controlled study**

**Version No: 2.9**

**PRINCIPAL INVESTIGATOR**

**Kyung-Il Park**

Professor

Department of Neurology,

Seoul National University Hospital Healthcare System Gangnam Center.

**Study Summary**

| Study title | Impact of selective cyclooxygenase-2 inhibitor, celecoxib on cortical excitability and electrophysiological property in brain of healthy volunteer: Randomized, double-blind, placebo-controlled study |
| --- | --- |
| Principal investigator | Professor Kyung-Il Park, Department of Neurology |
| Financial support | Pfizer Inc. |
| Objective | To evaluate the impact of celecoxib on cortical excitability and electrophysiological properties of the brain in healthy humans by electroencephalography (EEG) and transmagnetic stimulation (TMS), as ample evidence based on animal models of epilepsy has demonstrated that celecoxib has anticonvulsant effects.  [Study 1]  1) Primary object  - To evaluate the change of cortical excitability and electrophysiological properties in brain cortex to single oral dose of celecoxib (400mg once) in healthy volunteer  - To evaluate the change of cortical excitability and electrophysiological properties in brain cortex by long-term treatment of celecoxib (200mg twice-daily for 7 days) in healthy volunteer  2) Secondary object  - To investigate the electroencephalographic changes by celecoxib in different locations and different frequency band.)  [Study 2]  1) To evaluate the change of cortical excitability and electrophysiological properties in brain cortex by long-term treatment of celecoxib (200mg twice-daily for 7 days) in healthy volunteer |
| Study design | Phase 1, randomized, placebo-controlled, double-blind study |
| Study period | Date of IRB approval ~ 2017.9.30. |
| Study subjects | Inclusion criteria  1. Healthy male or female aged between 20 and 50 years.  2. Body mass index between 16.0 and 30.0 kg/m^2^.  Exclusion criteria  1. History of cardiovascular disease (heart disease, stroke)  2. History of hepatic disease  3. History of gastrointestinal hemorrhage or inflammatory bowel disease  4. History of seizure (epilepsy)  5. History of any kind of medication within 1 week before screening  6. History of hypersensitivity to any medication  7. Females who were pregnant, breastfeeding, or intended to become pregnant  8. Presence of clinically significant electrocardiogram abnormality at screening  9. Aspartate aminotransferase (AST) or alanine aminotransferase (ALT) greater than 2.0 × the upper normal limit  10. Serum creatinine levels greater than 1.5 × the upper normal limit  11. Hyperkelamia, K level ≥ 5.5 mmol/L  12. Galactose intolerance |
| Number of study subject | A total of 40 subjects  Study 1 (EEG study) - Celecoxib: 10 subjects/ placebo: 10 subjects  Study 2 (TMS study) - Celecoxib: 10 subjects/ placebo: 10 subjects |
| Vulnerable subjects | Employees |
| Study methods | Celecoxib  - Dose: 400mg/day  - Route of administration: oral  - Duration of administration: 7 days  Study 1  - After randomization, EEG will be performed at baseline just before celecoxib (400 mg) or placebo delivery at 9 AM. EEG will be repeated 4 hours after the administration. The subjects will take celecoxib or placebo 200 mg twice a day for 7 subsequent days, and a third EEG will be conducted 4 hours after the final dose.  Study 2  - After randomization, TMS will be performed at baseline just before celecoxib or placebo (200 mg) delivery. The subjects will take 200 mg of celecoxib or placebo twice a day for 7 days. The second TMS recording will be performed 4 hours after the final dose. |
| Efficacy evaluation | Study 1  Primary end point  - Change of power spectra by single high dose of celecoxib.  Secondary end point  - Change of power spectra by 7-day treatment.  - Change of electroencephalographic properties by celecoxib in specific locations or frequency band by single dose or 7-day treatment.  Study 2  Primary end point  - Change of resting motor potential, amplitude of MEP, cortical silent period, intracortical inhibition and intracortical facilitation by 7-day treatment of celecoxib |
| Adverse effect evaluation | Adverse effect or serious adverse effect  All the adverse events including status changes after enrollment will be recorded, regardless of causal relationship to study drug or examination.  The subjects will be observed directly for 30 minutes after taking study drug for immediate reactions. The subjects also will be observed during TMS procedure and until 30 min after examination. The subjects will be asked if they had any pain (by Visual analogue scale) immediately after TMS. The occurrence and severity of any adverse events by study drug or device during this visit will be recorded on the appropriate CRF |
| Expected result | The knowledge from this research will deepen our understanding about inflammation and neuronal activity and will be a basis of clinical study for utilizing celecoxib as an anticonvulsant in the future. |

**Table of Contents**

1. Title of the study ------------------------------------------------------------------------7

2. Research institution ---------------------------------------------------------------------7

3. Principal investigator and co-investigators --------------------------------------------7

1) Principal investigator

2) Co-investigator

3) Sub-investigator

4) Pharmacists

4. Sponsor ---------------------------------------------------------------------------------7

5. Financial support------------------------------------------------------------------------7

6. Study duration --------------------------------------------------------------------------7

7. Study subjects --------------------------------------------------------------------------8

8. Background and objectives ------------------------------------------------------------8

1) Background

2) Hypothesis and objectives

9. Study drug -----------------------------------------------------------------------------11

10 Eligibility criteria and sample size estimation ---------------------------------------11

1) Inclusion criteria

2) Exclusion criteria

3) Sample size estimation

4) Method of recruitment of subjects

11. Study methods -----------------------------------------------------------------------13

1) Detailed study methods ------------------------------------------------------------13

2) Subject group and randomization -------------------------------------------------14

3) Dose and route of administration of the study drug -----------------------------14

4) Items of observation and methods ------------------------------------------------15

5) Analysis -----------------------------------------------------------------------------17

6) Point of distinction -----------------------------------------------------------------18

7) Risk and benefit of subjects -------------------------------------------------------18

8) Dropout criteria --------------------------------------------------------------------18

9) Adverse event ----------------------------------------------------------------------19

10) Data and Safety Monitoring Plan ------------------------------------------------21

11) Method of analysis ---------------------------------------------------------------21

12) Study schedule -------------------------------------------------------------------22

12. Ethical consideration ----------------------------------------------------------------22

13. Human derived material ------------------------------------------------------------23

14. References ---------------------------------------------------------------------------23

**Study protocol**

1. **Title of the study**

Impact of a selective cyclooxygenase-2 inhibitor, celecoxib, on cortical excitability and electrophysiological properties of the brain in healthy volunteers: A randomized, double-blind, placebo-controlled study

1. **Research institution**

Seoul National University Hospital

101, Daehak-ro, Jongno-gu, Seoul, Republic of Korea

1. **Principal investigator and co-investigators**
2. Principal investigator: Prof. Kyung-Il Park, Department of Neurology, Seoul National University Hospital
3. Co-investigator

Prof. Sang Kun Lee, Department of Neurology, Seoul National University Hospital

Prof. Kon Chu, Department of Neurology, Seoul National University Hospital

Prof. Ki-Young Jung, Department of Neurology, Seoul National University Hospital

Prof. Keun-Hwa Jung, Department of Neurology, Seoul National University Hospital

Mi-Young Jung, Department of Neurology, Seoul National University Hospital

Il-Jung Kim, Department of Neurology, Seoul National University Hospital

1. Sub-investigator: Jin-Sun Jun Department of Neurology, Seoul National University Hospital
2. Pharmacists: Hongwon Jang, Mina Kim, Youjung Kim, Jinah Lee, Jandee Lee, Juyeon Lee
3. **Sponsor**1) Sponsor (name address): N/A
   2) Clinical research associate: N/A
4. **Financial support**

Pfizer Inc.

1. **Study period**

Date of IRB approval ~2017.9.30

1. **Study subject**

Healthy male or female aged between 20 and 50 years

1. **Background and objectives**

**1) Background**

The inflammatory response is considered a defence mechanism against physical or infectious insults, and it is prevalent within the central nervous system. In addition to brain injuries such as trauma, ischemia, or hypoxia, seizures result in a robust inflammatory cascade leading to enhanced activation of excitatory synaptic networks. Activation of glia and neurons by seizure rapidly induces a variety of pro-inflammatory cytokines, including cyclooxygenase-2 (COX-2).

Celecoxib is a highly selective inhibitor of cyclooxygenase-2 (COX-2) and a widely prescribed anti-inflammatory drug to alleviate the pain related to degenerative or rheumatoid arthritis. In addition to approved usage relating to its anti-inflammatory mechanisms of action, celecoxib has also been studied for repurposing as an anticancer drug as well as for the treatment of epilepsy.

Celecoxib has been studied in several in vivo epilepsy models, including acute seizures spontaneous recurrent seizures (SRS), and kindling models. The majority of studies have indicated anticonvulsant or neuroprotective effects of celecoxib, though a few studies have reported conflicting results. However, there is no study on the electrophysiological effect of celecoxib in humans.

◎ Previous study

| Celecoxib (10uM )suppresses neuronal excitability and ongoing seizures in *in vitro* model of epilepsy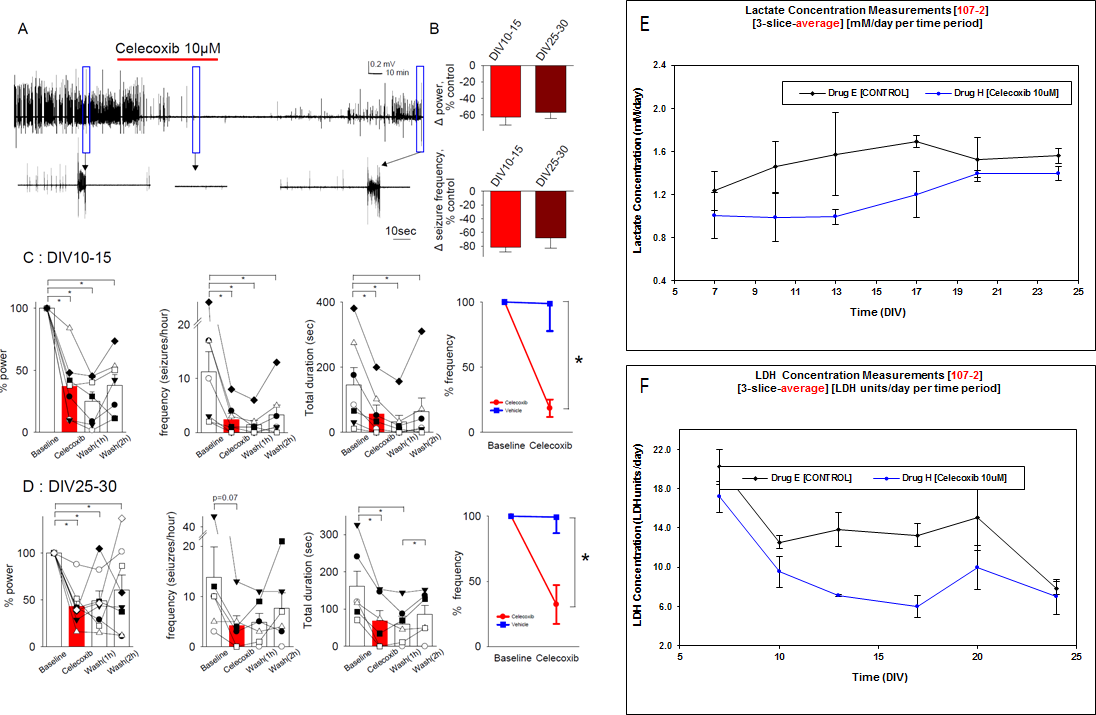  (A-D) In organotropic hippocampal slice model, celecoxib showed anticonvulsive effect.. (E,F) Celecoxib inhibited lactate and lactate dehydrogenase production. These indicate anticonvulsive and neuroprotective effects of celecoxib. (Park & Staley. unpublished data) |
| --- |

| In pilocarpine epilepsy model, celecoxib (20mg/kg) reduced spontaneous recurrent seizures.  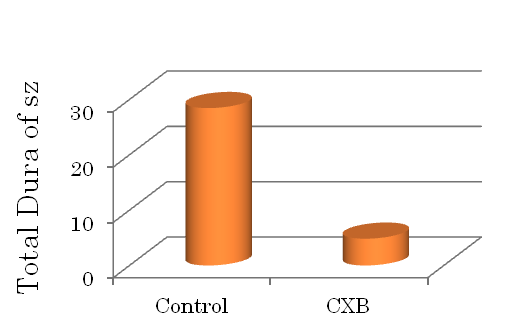 |
| --- |

◎ Pharmacodynamics of celecoxib

Total plasma concentration of celecoxib reach to maximum in 24 hours after oral concentration and area under the curve (AUC) is dose proportional. Celecoxib is eliminated predominantly by hepatic metabolism (CYP2C9) and the primary metabolite in both urine and feces as carboxylic acid and its glucuronide conjugate, with little unchanged drug recovered in urine and feces. Half-life elimination is 11 hours. Maximum plasma and CSF concentration after a single oral dose of celecoxib are similar, about in 2-4 hours.


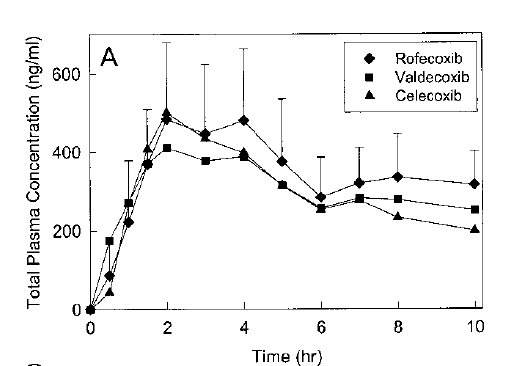

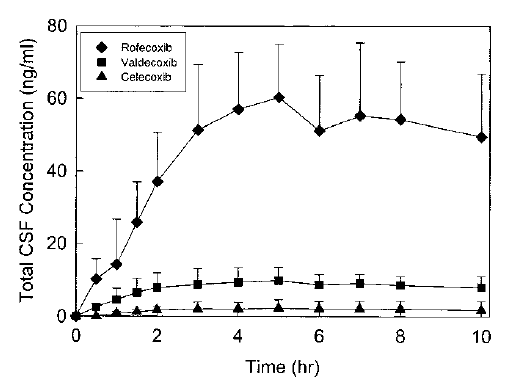


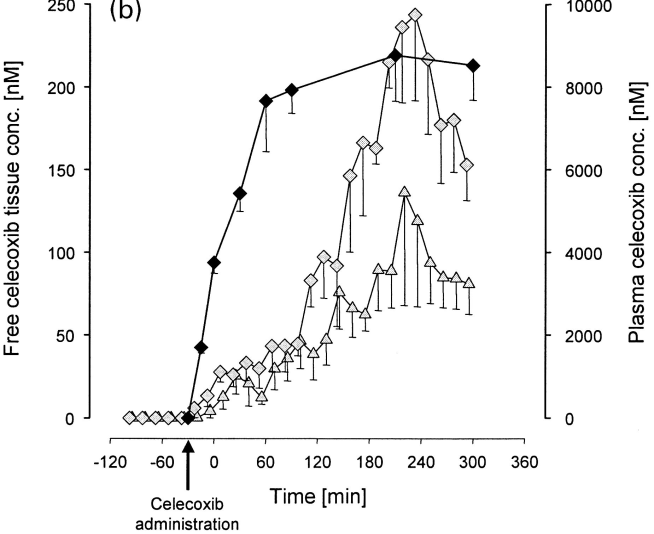


In brain tissue, concentration of celecoxib reach to maximum after 2-4 hours of oral administration.

◎ In this study, the volunteers take celecixob for 7 days, which is broadly used and safety has been proven. EEG and TMS will be performed before and after administration of study drug or placebo, and the exams are also safe and broadly used in clinical setting. As the study protocol is simple and safe, the outcome might be derived rapidly.

**2) Hypothesis and objectives**

To evaluate the impact of celecoxib on cortical excitability and electrophysiological properties of the brain in healthy humans by electroencephalography (EEG) and transmagnetic stimulation (TMS), as ample evidence based on animal models of epilepsy has demonstrated that celecoxib has anticonvulsant effects.

◎ Primary object

- To evaluate the change of cortical excitability and electrophysiological properties in brain cortex to single oral dose of celecoxib (400mg once) in healthy volunteer

- To evaluate the change of cortical excitability and electrophysiological properties in brain cortex by long-term treatment of celecoxib (200mg twice-daily for 7 days) in healthy volunteer

◎ Secondary object

- To investigate the electroencephalographic changes by celecoxib in different locations and different frequency band.

1. **Study drug (generic name and brand name), dosing, dosage forms**

◎ Study drug

- Generic name: Celecoxib

- Brand name: Celebrex^®^

- Dosing and dosage forms: 200mg capsule

◎ Medical device

- EEG

Conventional EEG (10-20 system), (Grass-Telefactor, USA)

- Motor evoked potential (transcranial magnetic stimulation. TMS)

Magstim 200 devices (Magstim Co. Ltd., Dyfed, S. Wales, UK).

A figure-of-eight shaped magnetic stimulator (type 9925, maximal output 2.2 Tesla)

1. **Eligiblity criteria of subjects and sample size estimation**

**1) Inclusion criteria**

1. Healthy male or female aged between 20 and 50 years.

2. Body mass index between 16.0 and 30.0 kg/m^2^

**2) Exclusion criteria**

1. Healthy male or female aged between 20 and 50 years.

2. Body mass index between 16.0 and 30.0 kg/m2.

Exclusion criteria

1. History of cardiovascular disease (heart disease, stroke)

2. History of hepatic disease

3. History of gastrointestinal hemorrhage or inflammatory bowel disease

4. History of seizure (epilepsy)

5. History of any kind of medication within 1 week before screening

6. History of hypersensitivity to any medication

7. Females who were pregnant, breastfeeding, or intended to become pregnant

8. Presence of clinically significant electrocardiogram abnormality at screening

9. Aspartate aminotransferase (AST) or alanine aminotransferase (ALT) greater than 2.0 × the upper normal limit

10. Serum creatinine levels greater than 1.5 × the upper normal limit

11. Hyperkelamia, K level ≥ 5.5 mmol/L

12. Galactose intolerance

**3) Sample size estimation**

◎ Dose (200mg or 400mg) justification: In the previous study, the CSF concentration of celecoxib (200 mg) 4 hours after oral administration was 2 ng/ml (= 5 nM), and the Ki was 3.7 nM. As the tissue concentration peaks at 4 hours, 200 mg of celecoxib is sufficient to evaluate its effects. However, most subjects in the study are Caucasian, and genetic polymorphism of CYP2C9 varies from other racial and ethnic populations. In addition, the Ki value could not be directly applied to the electrophysiological study. Therefore, more doses of celecoxib are needed to evaluate the acute effect of celecoxib on EEG power spectra. In previous clinical trial of celecoxib with colorectal cancer patients, administration of 400 mg of celecoxib twice a day did not increased adverse effect compared to the administration of 200 mg of celecoxib twice a day. Therefore, single dose (400 mg) of celecoxib would be safe to healthy volunteers.

◎ No similar previous study has investigated the effect of celecoxib on brain activity. Based on a previous paper evaluating cortical excitability using the same method and a different drug (Joo, 2008), the mean difference was 0.4 and the standard deviation of each group was 0.4 and 0.2. The formula used to calculate the sample size was a two-sided paired or unpaired test. The significance level and power were set to 5%, and 0.8, respectively. Based on the calculation (OpenEPi (ver3)), n=10 in each group was appropriate for this study. Because this study has 4 groups, 40 subjects should be enrolled.

**4) Method of recruitment of subjects**

◎ On the bulletin board of Seoul National University or Seoul National University Hospital

◎ Prints on newspapers

1. **Study methods**
2. **Detailed study methods**

After screening test through blood lab, the subject will be included and randomized to each group as Table 1 if they met inclusion and exclusion criteria and agree informed consent.

**Table 1.**

| **Study 1** | **Study 2** |
| --- | --- |
| Celecoxib 1 group **(n = 10)** | Celecoxib 2 group **(n = 10)** |
| Placebo 1 group **(n = 10)** | Placebo 2 group **(n = 10)** |

**Study 1 (Figure)**

EEG will be performed at baseline just before celecoxib (400 mg) or placebo delivery at 9 AM, and EEG will be repeated at 1 PM, 4 hours after administration To evaluate the long-term effects of celecoxib, subjects will take celecoxib or placebo 200 mg twice a day for 7 subsequent days, and a third EEG will be conducted at 1 PM on the last day, 4 hours after the final dose

**
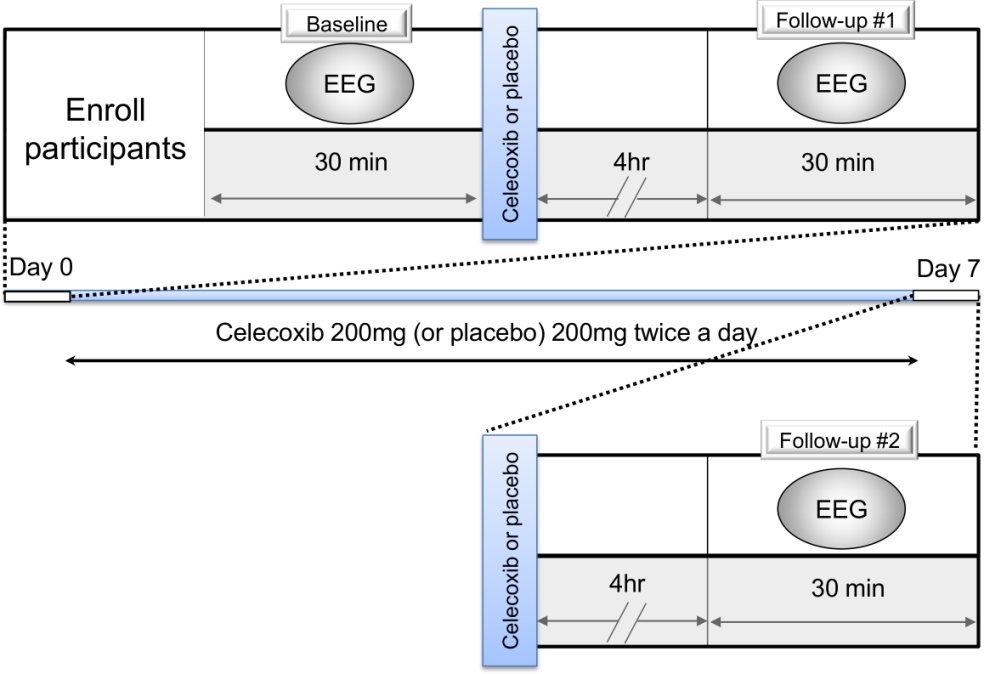
**

**Study 2 (Figure)**

TMS will be performed at baseline just before celecoxib or placebo (200 mg) delivery. The subjects will take 200 mg of celecoxib or placebo twice a day for 7 days. The second TMS recording will be performed 4 hours after the final dose

**
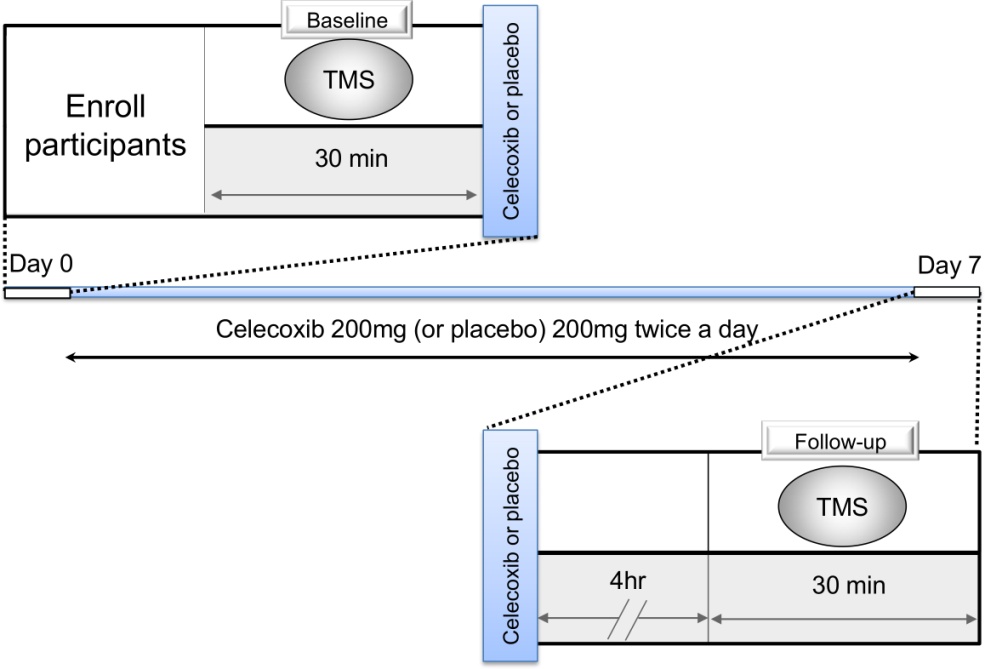
**

EEG: electroencephalography; TMS: transmagnetic stimulation

**2) Subject group and randomization**

This study is a double-blind, randomized placebo-controlled trial to reduced selection bias, allocation bias, balancing both known and unknown characteristics of the subjects, and maximize statistical power. The allocation assignment will be not opened during tue study to investigators and subjects. Online software (Research randomizer at https://www.randomizer.org) will generate randomization code and Pfizer Inc. will keep the Randomization Code/list in a secure manner. Blinding codes will be broken after completing all procedures of all targeted population. However, investigator might request the Log to be opened if s/he anticipates a real possibility of adverse events related to the investigational agents in urgent situation. Any request to break a randomization must be put into writing; the time, date, and reason of request must be documented with CRF

**3) Dose and route of administration of the study drug**

Study 1: celecoxib (400 mg) or placebo once on the first day, celecoxib or placebo 200 mg twice a day for 6 subsequent days and celecoxib or placebo 200 mg once on the last day, oral dministration

Study 2: celecoxib or placebo 200 mg twice a day for 7 days and celecoxib or placebo 200 mg once on the last day, oral dministration

**4) Items of observation and methods**

**Study**

◎ Epidemiologic data

- Age, date of birth, age

- Medication history

- Medical history

- Physical examination

◎ Laboratory data

- Blood lab: AST/ALT, serum creatinine, potassium

- EKG

**-** EEG

- According to the international 10-20 system for 15 miniutes

- EEG (power spectra analysis)

The EEG data will be bandpass filtered (0.5-50 Hz) after re-referenced to the common average reference for analysis, and 10 epochs of 1.5 seconds in duration in the eyes-closed state without artefacts will be chosen. For power spectral analysis, the electrodes will grouped into frontal, central and parietal regions and analyzed by band width.

| Primary end point  - Change of power spectra by single high dose of celecoxib.  Secondary end point  - Change of power spectra by 7-day treatment.  - Change of electroencephalographic properties by celecoxib in specific locations or frequency band by single dose or 7-day treatment. |
| --- |

**Study 2**

◎ Epidemiologic data

- Age, date of birth, age

- Medication history

- Medical history

- Physical examination

◎ Laboratory data

- Blood lab: AST/ALT, serum creatinine, potassium

- EKG

- Motor evoked potential (MEP)

- A figure-of-eight shaped magnetic stimulator will be located over the contralateral motor cortex to produce optimal motor potentials in the first dorsal interosseous muscle.

1) The resting motor threshold (RMT): the lowest intensity required to evoke MEPs in the FDI with a peak-to-peak amplitude of at least 50 μV in 4 of 8 trials

2) Peak-to-peak amplitude: With the FDI muscle in the relaxed state, stimuli will be delivered to evoke MEPs at stimulus intensities of 120%, 140%, and 150% (if possible) of RMT. [16]. Eight stimuli will be delivered randomly at each stimulus intensity every 5 seconds, and the average of the peak-to-peak amplitudes will be measured

3) Cortical silent period: The time between the end of the stimulus-induced MEP and the reoccurrence of voluntary EMG activity,

| Primary end point  - Change of resting motor potential, amplitude of MEP, cortical silent period, intracortical inhibition and intracortical facilitation by 7-day treatment of celecoxib |
| --- |

| **Study 1** | Screening  (Visit 1) | Visit 2(1^st^ day) | 2^nd^ day ~ 6^th^ day | Visit 3 (7^th^ day) |
| --- | --- | --- | --- | --- |
| Informed consent | V |  |  |  |
| Age, date of birth, age | V |  |  |  |
| Medication history | V |  |  |  |
| Medical history | V |  |  | V |
| Physical examination | V |  |  |  |
| Blood lab | V |  |  |  |
| EKG | V |  |  |  |
| Study drug administration |  | V | V | V |
| Adverse event |  | V |  | V |
| EEG |  | V (2 times) |  | V(once) |

| **Study 2** | Screening  (Visit 1) | Visit 2(1^st^ day) | 2^nd^ day ~ 6^th^ day | Visit 3 (7^th^ day) |
| --- | --- | --- | --- | --- |
| Informed consent | V |  |  |  |
| Age, date of birth, age | V |  |  |  |
| Medication history | V |  |  |  |
| Medical history | V |  |  | V |
| Physical examination | V |  |  |  |
| Blood lab | V |  |  |  |
| EKG | V |  |  |  |
| Study drug administration |  | V | V | V |
| Adverse event |  | V |  | V |
| MEP |  | V(once) |  | V(once) |

**5) Analysis**

◎ Analysis

- Per protocol (if needed intention to treat will be used)

◎ EEG power spectra analysis

- 1^st^ EEG vs 2^nd^ EEG: to evaluate acute effect of celecoxib administration.

- 1^st^ EEG vs 3^rd^ EEG: to evaluate long-term effect of celecoxib administration

Variables between subjects (celecoxib vs. placebo) and within subject (1^st^ EEG vs 2^nd^ EEG, 1^st^ EEG vs 3^rd^ EEG) will be analyzed

◎ TMS analysis

- Before and after of 7-days of celecoxib administration. Variables between subjects (celecoxib vs. placebo) and within subject (before vs. after) will be analyzed

**6) Point of distinction**

◎ Celecoxib has been studied in several in vivo epilepsy models, including acute seizures, spontaneous recurrent seizures, and kindling models. The majority of studies have indicated anticonvulsant or neuroprotective effects of celecoxib. However, there is no study on the electrophysiological effect of celecoxib in humans. This research will deepen our understanding about inflammation and neuronal activity and will be a basis of clinical study for utilizing celecoxib as an anticonvulsant in the future.

**7) Risk and benefit of subjects**

◎ Study drug

- Refer to instructions for detailed side effects. This medication is widely used to treat degenerative and rheumatoid arthritis, ankylosing spondylitis, and acute pain. In the large clinical trials before FDA approval and post market surveillance, stomach upset, facial swelling, abdominal pain, nausea, rash, diarrhea, insomnia may occur. However, in a study with 12 subjects, there was no serious side effect with 2400mg oral administration. As the study subjects are heathy adults, and the study drug is FDA approved and the dose of study drug is tolerable, the possibility of occurrence of side effect is very low. All study subject will be insured for in case of having any adverse events.

◎ Exams

- EEG is painless and safe.

- rTMS - rTMS is well-tolerated and associated with few side-effects. The common side effect is headahes but these are mild and generally diminish spontaneously.

**8) Dropout criteria**

Dropout could be decided at any time during the study period for the following reasons. If the patient had any drinks containing caffein (coffee, green tea, or energy drink) or took alcohol, when the subject does not wish to proceed with study drug administration or withdraws consent during the study period, in the cases where the subject requires to discontinue the investigational product or any case in which serious adverse event (SAE) or adverse drug reaction is observed, poor study drug compliance (<80%), when the subjects could not be undergone rTMS due to headache. the subject takes any medication that is expected to influence the safety and pharmacokinetic characteristics of investigational product, without permission of the investigator, when the major protocol deviation occurs such as subject found not to be eligible based on inclusion and exclusion criteria after enrollment.

**9) Adverse events (AEs)**

**◎ Definition of AEs**

An adverse event is defined as any unfavorable and unintended change in the structure, function, or chemistry of the body, or worsening of a pre-existing condition, temporally associated with the use of a product whether or not considered related to the use of the product or device. In this study, such changes will be monitored, and summarized.

**◎ Definition of Serious AEs**

ICH (International Conference on Harmonization) defined serious AE as below:

- resulted in death or is life-threatening,

- required inpatient hospitalization or prolongation of existing hospitalization,

- resulted in persistent or significant disability/incapacity,

- resulted in a congenital anomaly/birth defect.

**◎ Assessment method for severity of AE**

| 1 = Mild | Awareness of signs or symptoms, but easily tolerated; is of minor irritant; causing no loss of time from normal activities |
| --- | --- |
| 2 = Moderate | Discomfort severe enough to cause interference with usual activities |
| 3 = Severe | Incapacitating with inability to work or do usual activity |

**◎ Causality assessment**

| Causality | Assessment criteria |
| --- | --- |
| Not related | Event before taking the drug  Event or laboratory test abnormality, with a time to drug intake that makes a relationship improbable  Definite other plausible explanations |
| Doubtful | Event or laboratory test abnormality, with a time to drug intake that makes a relationship improbable (but not impossible)  Disease or other drugs provide plausible explanations |
| Possible | Event or laboratory test abnormality, with reasonable time relationship to  drug intake  Could also be explained by disease or other drug  Information on drug withdrawal may be lacking or unclear |
| Probable | Event or laboratory test abnormality, with reasonable time relationship to  drug intake  Unlikely to be attributed to disease or other drugs  Response to withdrawal clinically reasonable  Rechallenge not required |
| Very likely | Event or laboratory test abnormality, with plausible time relationship to  drug intake  Cannot be explained by disease or other drugs  Response to withdrawal plausible (pharmacologically, pathologically)  Event definitive pharmacologically or phenomenologically (i.e. an  objective and specific medical disorder or a recognised pharmacological  phenomenon)  Rechallenge satisfactory, if necessary |

**◎ Action taken**

0 = No action taken

1 = Temporary discontinuation of study drug administration

2 = Discontinuation of study drug administration

3 = Medical intervention

4 = Non-medical intervention

5 = Hospitalization

**◎ Report of AE**

At Visit 2, Study personnel will directly observe study subjects for 30 minutes after taking study drug for immediate reactions. The subjects also observed during TMS procedure and until 30 min after examination. Study personnel will ask the degree of pain (Visual analogue scale) immediately after TMS. The occurrence and severity of any AE by study drug or device during this visit will be recorded on the appropriate CRF. The subjects will be asked to note and characterize in their own words about any local or systemic AEs they experience, during 7-day period between visit 2 and 3.

At visit 3, study subjects will be queried regarding the occurrence of any SAE or other unexpected AE that may have occurred since the last visit. These events will be recorded on the CRFs.

The reporting period for AEs is the period starting from the time of administration of study drug to the day of study completion (visit 3).

Events that involve a death or are unexpected, serious and related or possibly related to the investigational drug will be submitted to the Pfizer Inc. within 24 hours of discovery. In case blinding code inevitably needs to be broken for treatment of subject, investigator must report it to the Pfizer Inc. within 24 hours of discovery.

**10) Data and Safety Monitoring Plan**

The principle investigator (an authorized investigator in the absence of the principle investigator) is designated as personnel for data security, and warrants the completeness of the data by collecting and reviewing the data on recruiting every 10 subjects and monitoring about constant safety, and establishes the safety of the study subjects.

**11) Method of analysis**

**Study1**

◎ Change of EEG power spectra by single dose or 7-day treatment of celecoxib or placebo

- Variables between subjects (celecoxib vs. placebo) and within subject (1^st^ EEG vs 2^nd^ EEG, 1^st^ EEG vs 3^rd^ EEG) will be analyzed by student-t test and paired-t test. Analysis of EEG data will consider frequency bands (alpha, delta, theta and beta) and locations (frontal, central and parietal) using repeated measures analysis of variance (rmANOVA). A two-sided 5% significance level will be used for all statistical differences.

**Study 2**

◎ Change of rTMS variables by 7-day treatment of celecoxib or placebo

- Regarding TMS parameters, paired-t-test or Wilcoxon’s signed rank test will be used. RmANOVA will be used for comparison of TMS variables between celecoxib and placebo. A two-sided 5% significance level will be used for all statistical differences.

1. **Study schedule**

|  | Befor May | May | Jun | Jul | Aug | Sep | Oct | Nov | Dec |
| --- | --- | --- | --- | --- | --- | --- | --- | --- | --- |
| - IRB approval | V |  |  |  |  |  |  |  |  |
| - FDA approval | V |  |  |  |  |  |  |  |  |
| - Recruitment and screening |  | V | V | V | V | V | V |  |  |
| - Study |  |  | V | V | V | V | V | V |  |
| - Analysis |  |  |  |  |  |  |  | V | V |
| - Result |  |  |  |  |  |  |  |  | V |

**12. Ethical consideration**

1. **Basic principal**

◎ Principle investigator

- This study will be conducted in accordance with Korea Good Clinical Practice (KGCP) and the principles of ‘The Declaration of Helsinki’. ICH Guideline E2A ‘Clinical Safety Data Management: Definitions and Standards for Expedited Reporting’ will also be complied with

◎ Institutional review board

- For medical research using changed protocol, new protocol need to be approved by the institutional review board (IRB). Before the approval of IRB, the trial cannot start of recruitment.

1. **Informed consent**

Participation by individuals capable of giving informed consent as subjects in medical research must be voluntary. Before the enrollment, each potential subject must be adequately informed of the aims, methods, expected results and safety.

1. **Payment for subjects**

To enrolled patient who met inclusion/exclusion criteria and signed voluntary written informed consent, 150000 will be paid after end of the study.

1. **Private information of subjects**

Patient’s identification data will be will be stored safely at the principal investigator’s independent storage and kept separate from the data set used for analysis. All participants’ private information will be protected before and after the publication.

As the study progresses, adherence to instrument and intervention manuals will be continuously monitored and training refreshed by the trial staff responsible for data collection and application of the intervention.

1. **Protecting vulnerable subjects**

An employee’s decision to participate in, not to participate in, or to withdraw from employer-based research have no effect on the employee’s hiring, promotion, performance evaluation, or other employment benefits.

**13. Human derived material**

N/A

**14. Reference**

Bertagnolli MM, Eagle CJ, Zauber AG, Redston M, Solomon SD, Kim K, Tang J, Rosenstein RB, Wittes J, Corle D, Hess TM, Woloj GM, Boisserie F, Anderson WF, Viner JL, Bagheri D, Burn J, Chung DC, Dewar T, Foley TR, Hoffman N, Macrae F, Pruitt RE, Saltzman JR, Salzberg B, Sylwestrowicz T, Gordon GB, Hawk ET; APC Study Investigators. Celecoxib for the prevention of sporadic colorectal adenomas. N Engl J Med. 2006 Aug 31;355(9):873-84.

Dembo G, Park SB, Kharasch ED. Central nervous system concentrations of cyclooxygenase-2 inhibitors in humans. Anesthesiology. 2005 Feb;102(2):409-15.

Oliveira MS, Furian AF, Royes LF, Fighera MR, Fiorenza NG, Castelli M, Machado P, Bohrer D, Veiga M, Ferreira J, Cavalheiro EA, Mello CF. Cyclooxygenase-2/PGE2 pathway facilitates pentylenetetrazol-induced seizures. Epilepsy Res. 2008;79(1):14-21.

Jung KH, Chu K, Lee ST, Kim J, Sinn DI, Kim JM, Park DK, Lee JJ, Kim SU, Kim M, Lee SK, Roh JK. Cyclooxygenase-2 inhibitor, celecoxib, inhibits the altered hippocampal neurogenesis with attenuation of spontaneous recurrent seizures following pilocarpine-induced status epilepticus. Neurobiol Dis. 2006;23(2):237-46

Joo EY, Kim SH, Seo DW, Hong SB. Zonisamide decreases cortical excitability in patients with idiopathic generalized epilepsy. Clin Neurophysiol. 2008;119(6):1385-92.

Park KI, Dzhala V, Saponjian Y, Staley KJ. What elements of the inflammatory system are necessary for epileptogenesis in vitro? eNeuro March 3, 2015,

Park KI, Saponjian Y, Dzhala V, Mail M, Staley KJ. Anticonvulsant action of the cyclooxygenase inhibitor, Celecoxib in an in vitro chronic post-traumatic epilepsy model. Poster presentation at Society for Neuroscience meeting 2013.

Buysse DJ, Germain A, Hall ML, et al. EEG spectral analysis in primary insomnia: NREM period effects and sex differences. Sleep 2008;31:1673–82.

Perlis ML, Merica H, Smith MT, Giles DE. Beta EEG activity and insomnia. Sleep Med Rev 2001;5:363–74.

Jung KY, Koo YS, Kim BJ, Ko D, Lee GT, Kim KH, Im CH. Electrophysiologic disturbances during daytime in patients with restless legs syndrome: further evidence of cognitive dysfunction? Sleep Med. 2011;12(4):416-21.

Tegeder I, Niederberger E, Vetter G, Bräutigam L, Geisslinger G. Effects of selective COX-1 and -2 inhibition on formalin-evoked nociceptive behaviour and prostaglandin E(2) release in the spinal cord. J Neurochem. 2001 Nov;79(4):777-86.

Reyners AK, de Munck L, Erdkamp FL, Smit WM, Hoekman K, Lalisang RI, de Graaf H, Wymenga AN, Polee M, Hollema H, van Vugt MA, Schaapveld M, Willemse PH; DoCaCel Study Group. A randomized phase II study investigating the addition of the specific COX-2 inhibitor celecoxib to docetaxel plus carboplatin as first-line chemotherapy for stage IC to IV epithelial ovarian cancer, Fallopian tube or primary peritoneal carcinomas: the DoCaCel study. Ann Oncol. 2012 Nov;23(11):2896-902.
