# Supplementary material for: Impact of a selective cyclooxygenase-2 inhibitor, celecoxib, on cortical excitability and electrophysiological properties of the brain in healthy volunteers: A randomized, double-blind, placebo-controlled study
Source: PLoS One. 2019 Feb 22;14(2):e0212689. doi: 10.1371/journal.pone.0212689 (PMC6386435; doi:10.1371/journal.pone.0212689)
Supplement: S4 File — (DOCX) [file pone.0212689.s004.docx]

**선택적 cyclooxygenase2 억제제인 celecoxib이 건강인 뇌의 피질흥분성과 전기생리학적 성상에 미치는 영향 규명: 무작위, 이중눈가림, 위약대조군 연구**

**Impact of selective cyclooxygenase-2 inhibitor, celecoxib on cortical excitability and electrophysiological property in brain of healthy volunteer: Randomized, double-blind, placebo-controlled study**

**Version No: 2.9**

**책임연구자 소속: 서울대병원 신경과 (강남센터)**

**책임연구자 이름: 박경일**

**PRINCIPAL INVESTIGATOR**

**Kyung-Il Park**

Professor

Department of Neurology, Seoul National University Hospital Healthcare System Gangnam Center.

**연구 개요**

| 연구제목 (study title) | (국문) 선택적 cyclooxygenase2 억제제인 celecoxib이 건강인 뇌의 피질흥분성과 전기생리학적 성상에 미치는 영향 규명: 무작위 이중 눈가림, 위약대조군 연구 |
| --- | --- |
|  | (English) Impact of selective cyclooxygenase-2 inhibitor, celecoxib on cortical excitability and electrophysiological property in brain of healthy volunteer: Randomized, double-blind, placebo-controlled study. |
| 책임연구자 **(Principal investigator)** | 신경과 박경일 교수  Professor **Kyung-Il Park,** Department of Neurology |
| 연구비 지원기관 (Financial disclosure) | 화이자 제약 (Pfizer Inc.) |

| 연구 목적  (Objective) | 동물모델을 대상으로 한 선행연구에서 뇌전증 발작을 줄이는 효과가 입증된 celecoxib을 건강한 성인에 투여한 후 전기생리학 검사도구를 이용하여 뇌피질의 흥분성이 감소됨을 확인하고자 함.  [연구1]  1) 1차 목적  - Celecoxib 의 단회 투여에 의한 건강인의 뇌피질 흥분성 감소 효과 규명  - Celecoxib 의 1 주일간의 투여에 의한 건강인의 뇌피질 흥분성 감소 효과 규명  2) 2차 목적  -Celecoxib 투여 후, 뇌의 각 부위별, 뇌파의 파장별 변화 양상 규명  [연구2]  1) Celecoxib의 1주일간의 투여에 의한 건강인의 운동유발전위 (뇌피질흥분성)변화효과 규명 |
| --- | --- |
| 연구 설계 | 건강한 성인을 대상으로, 이중눈가림, 무작위배정, 위약 대조 방법으로, celecoxib의 단회 투여 또는 1주일간 투여하고, 전후 뇌파검사 또는 운동유발전위검사를 반복 시행하여 변화를 평가함. |
| 연구 기간 | IRB승인일~2017.9.30 |
| 연구 대상  (시험약 등) | 선정기준  1. 20세 이상 50세 이하의 남녀  2. 신체질량지수 16.0이상 30.0kg/m^2^이하  제외기준  1. 심장질환, 뇌졸중 등 심뇌혈관 질환자  2. 간 질환자  3. 위장출혈, 염증성 장 질환자  4. 발작과 뇌전증(간질)병력이 있는자  5. 최근 1주일 이내 모든 약물 복용력이 있는자  6. 약물 알러지 (두드러기, 안면부종, 쇼크 등) 병력이 있는자  7. 임신 중이거나 수유중인 자  8. 심전도의 임상적으로 유의미한 이상  9. AST/ALT ≥ 정상 상한치의 2배  10. 혈청 크레아티닌 ≥ 정상 상한치의 1.5배  11. 고칼륨혈증 ≥ 5.5 mmol/L  12. 갈락토오스 불내성 |
| 연구 대상자 수 | 총 40명  연구1(뇌파연구): 시험군: 10명 / 대조군 10명  연구2(운동유발전위연구): 시험군 10명 / 대조군: 10명 |
| 취약한 연구대상자 | 피고용자 |
| 연구 방법 | - 투여량: 1일 400mg  - 투여방법: 경구  - 투여기간: 7일  **연구1**  - 무작위 배정 후, 뇌파검사를 실시한다. 검사 직후, 시험약 400mg 또는 시험약과 같은 모양과 무게를 가진 위약을 복용한다. 복용 4시간 후 뇌파검사를 시행한 후 귀가한다  - 다음 날부터 6일간 시험약 200mg 또는 위약을 하루 두 번씩 복용한다.  - 7일째 아침에 시험약 또는 위약을 복용한 후 4시간후 뇌파검사를 다시 시행한다.  **연구2**  - 무작위 배정 후, 운동유발전위검사를 실시한다. 검사 직후, 시험 약 200mg 또는 시험약과 같은 모양과 무게를 가진 위약을 복용한다. 귀가한 후 저녁에 시험약 200mg 또는 위약을 복용한다.  - 다음 날부터 6일간 200mg 을 하루 두 번씩 복용한다  - 7일째 아침에 시험약 또는 위약을 복용한 후 4시간 후, 운동유발전위검사를 다시 시행한다. |
| 유효성 평가 | **연구 1**  일차 종점 (primary end point)  - 시험약 1회 단회 투여에 의한 뇌파 파워스펙트라의 변화  이차 종점 (secondary end point)  - 시험약 7일 투여에 의한 뇌파 파워스펙트라의 변화  - 시험약 7일 투여에 의한 뇌특정부위와 특정파장의 뇌파성질의 변화  **연구2**  일차 종점 (primary end point)  **-** 시험약 7일 투여에 의한 운동유발전위 항목 (resting motor potential, amplitude of MET, cortical silent period, intracortical inhibition and intracortical facilitation) 의 변화 |
| 안전성 평가 | **부작용 또는 심각한 부작용**  시험약 또는 위약복용 후, 또는 검사 후, 연구대상자에 생기는 모든 건강상의 변화를 포함한다. 약물이나 검사와 연관성여부에 상관없이 모든 변화를 기록.  초회 복용 후, 30분간 급성반응을 관찰  운동유발검사중과 30분후까지 관찰하며 통증여부를 평가  연구대상자가 7일간 약물 복용 중 발생하는 변화를 기록하도록 하며, 모든 내용은 증례기록지에 기록 |
| 기대효과 및  예상결과 | 건강인을 대상으로 celecoxib 의 단회 투여 또는 1주일간의 투여에 의해 뇌의 전기생리학적 변화, 즉 뇌흥분성 감소가 증명된다면, 이는 향후 뇌전증 환자의 치료제로서의 celecoxib 또는 유사 물질의 효과를 뇌전증 환자에서 발작억제효과 증명하는 임상 연구의 초석이 될 것임. |

**목 차**

1. 연구 제목 ------------------------------------------------------------------------7

2. 연구의 실시기관 명칭 및 주소 ---------------------------------------------------7

3. 연구책임자 및 공동연구자 성명 및 직명 ----------------------------------------7

1) 연구책임자

2) 연구담당자

3) 공동연구자

4) 임상시험용 의약품 관리약사

4. 연구 의뢰기관 -------------------------------------------------------------------7

5. 연구비 지원기관 명칭 및 주소 --------------------------------------------------7

6. 예상 연구기간 ---------------------------------------------------------------------7

7. 연구 대상 질환 ---------------------------------------------------------------------7

8. 연구 배경 및 목적----------------------------------------------------------------- 7

1) 연구 배경

2) 연구 가설 및 목적

9. 임상연구용 의약품 및 의료기기, 원료약품의 분량, 제형 -----------------------11

10 연구대상자의 선정 기준, 제외기준, 목표한 대상자 수 및 산출 근거----------11

1) 선정기준

2) 제외기준

3) 목표한 대상자 수 및 산출 근거

4) 연구 대상자 모집 계획

11. 연구 방법 -------------------------------------------------------------------------12

1) 구체적인 연구방법 -------------------------------------------------------------12

2) 비교군 설정 및 무작위 배정 방법----------------------------------------------14

3) 시험약 투여 사용량, 투여방법-----------------------------------------------------14

4) 관찰항목, 임상검사항목 및 관찰검사방법 ----------------------------------------14

5) 효과 평가기준, 방법-----------------------------------------------------------------15

6) 기존 연구와의 차별점 -------------------------------------------------------------16

7) 연구대상자의 이익과 위험---------------------------------------------------------16

8) 중지 탈락 기준---------------------------------------------------------------------17

9) 부작용을 포함한 안전성의 평가기준, 방법 및 보고 방법-----------------------17

10) 자료 안전성 모니터링 계획------------------------------------------------------20

11) 자료 분석 및 통계 분석 방법----------------------------------------------------20

12) 연구 수행 일정표-----------------------------------------------------------------21

12. 연구대상자의 안전보호를 위한 대책-----------------------------------------------21

13. 인체유래물의 보관 및 폐기 방법---------------------------------------------------23

14. 참고 문헌 ----------------------------------------------------------------------------23

**연구계획서**

1. **연구 제목**

선택적 cyclooxygenase2 억제제인 celecoxib이 건강인 뇌의 피질흥분성과 전기생리학적 성상에 미치는 영향 규명: 무작위, 이중눈가림, 위약대조군 연구

1. **연구의 실시기관 명칭 및 주소**

서울대학교병원, 서울특별시 종로구 대학로 101,

1. **연구책임자 및 공동연구자 성명 및 직명**
2. 연구책임자: 박 경 일, 교수, 서울대학교병원 신경과
3. 공동연구자: 이 상 건, 교수, 서울대학교병원 신경과

주 건, 부교수, 서울대학교병원 신경과

정 기 영, 부교수, 서울대학교병원 신경과

정 근 화 부교수, 서울대학교병원 신경과

정 미 영, 연구원, 서울대학교병원 신경과

김 일 중 연구원, 임상병리사

1. 연구담당자: 전 진 선 전임의, 서울대학교병원 신경과
2. 임상시험용 의약품 관리 약사: 장홍원, 김미나, 최유정, 이진아, 김잔디, 이주연, 의생명 연구원 중앙임상시험센터
3. **연구 의뢰기관**1) 연구 의뢰기관 명칭 및 주소: 해당 없음
   2) 모니터요원 성명 및 직명: 해당 없음
4. **연구비 지원기관 명칭 및 주소**

한국 화이자 제약

1. **예상연구기간**

IRB 승인일 이후 ~2017.9.30

1. **연구 대상 질환**

20세이상 50세이하의 건강한 성인

1. **연구의 배경 및 목적**

**1) 연구 배경**

◎ **염증반응**은 신체 모든 부위에서 나타나며, 외부의 기계적 자극 또는 감염원에 의해 나타나는 **인체 방어작용**으로 해석되고 있음. 일반적으로 조직의 울혈과, 부종, 통증 등으로 나타나며, T-cell, B-cell, natural killer cell, 단핵구 계통의 대식세포 등이 동원되어 서로 synergistic reaction 을 일으키게 되는데 뇌의 자극 또는 손상 이후에도 다른 신체조직과 마찬가지로 innate immunity 와 adaptive immunity가 모두 관여하게 됨.

◎ 염증반응의 폭발적인 증폭에는, 신경세포(neuron)와 미세아교세포(microglia)에서의 cyclooxygenase-2 의 분비증가가 중요한 역할을 한다고 알려져 있음.

◎ 뇌의 물리적 손상 (뇌외상, 허혈, 저산소증 등), 뇌감염 뿐 아니라, **발작과 같은 과도한 전기적 흥분상태도 뇌의 염증반응을 유발**시키고 촉진시키는데, 이는 다른 뇌세포의 자극과 추가적인 손상을 일으키게 되어 흥분성 시냅스의 증가로 이어지며, 이는 뇌전증발생의 한 기전으로 설명되고 있음.

◎ Celecoxib은 선택적 cyclooxygenase-2의 억제제로서, 뇌내 염증반응시 그 분비가 증가되는 핵심단백질이면서, **여러 동물모델에서 뇌전증 발작을 억제**한다는 이전 연구결과가 많아서, 뇌전증치료제로서 가능성을 주목 받아왔음.

◎ 소염진통제로 광범위하게 쓰이는 celecoxib은 소염 진통효과 이외에도 최근 유방암, 대장암 등의 항암제의 추가요법으로 그 효과가 있음이 알려져 있는 등, 다양한 질환에 새로운 적응증이 있을 가능성이 높음.

◎ 동물실험 결과, celecoxib 은 뇌세포의 보호효과와 함께, 뇌파의 비정상적인 흥분현상인 ‘발작’ 억제효과도 있음. 정상인을 대상으로 본 연구가 성공적으로 시행되어 본 약제의 뇌 전기현상에 끼치는 영향을 처음으로 관찰하게 된다면, 이는 **향후 뇌전증 환자의 치료제로서의 효과를 규명하는 연구의 초석**이 되는 매우 중요한 연구가 될 것임.

◎ 선행 연구 결과


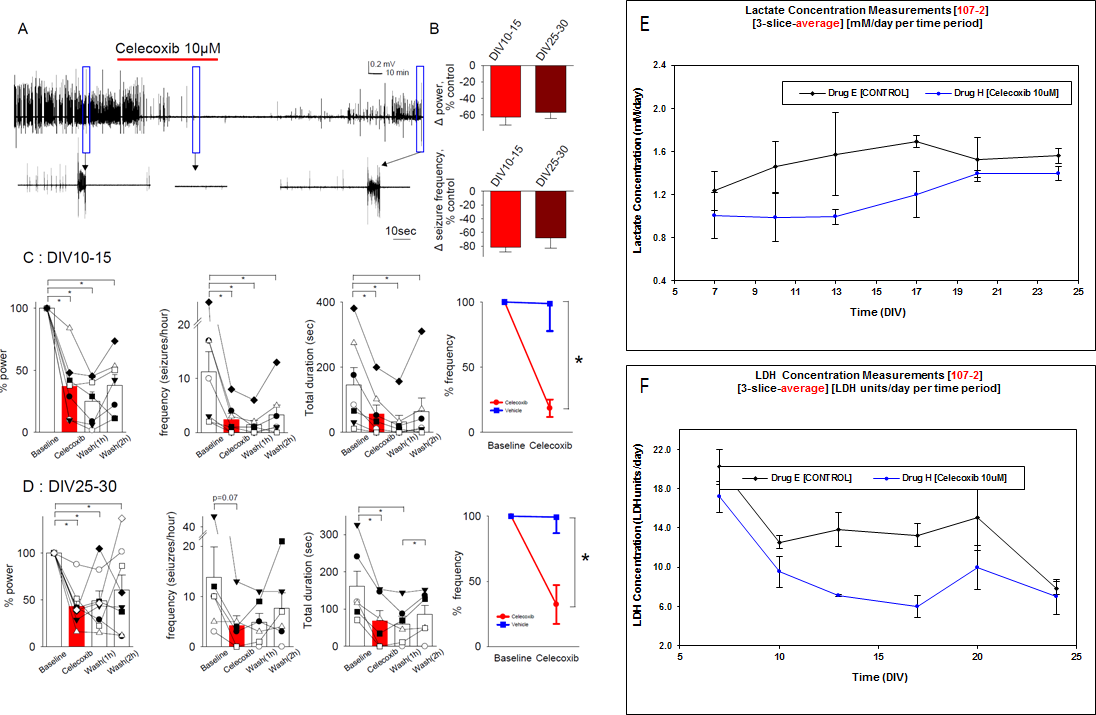
기관형 해마절편 뇌전증 모델에서 celecoxib (10uM) 의 급성 발작억제 효과

(A-D) 해마절편모델에서 cyclooxygenase-2 억제제인 celecoxib 의 발작억제. (E,F) celecoxib 에 의해 젖산과 젖산탈수소효소(lactate dehydrogenase)감소는 celecoxib 의 항경련효과와 뇌세포보호효과를 시사함. (Park & Staley. unpublished data)

필로칼핀 뇌전증모델에서 celecoxib (20mg/kg) 의 뇌전증 억제효과


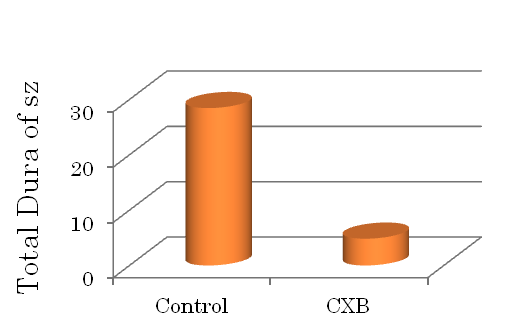


◎ Celecoxib 의 약물역동학

경구투여 시 24시간 내에 혈장 최고농도에 이르며, 먹는 용량에 비례하여 AUC가 증가함. Carboxylic acid 와 glucuronide 대사물질에 의해 대사된 후, 제거되며 극소량은 unchanged form 으로 대변이나 소변을 통해 배설된다. 제거반감기는 11시간이고, CYP2C9 에 의해 대사됨. 경구 단회 투여 (200mg) 후 혈장농도는 2-4시간에 최고농도에 이르며, 뇌척수액농도도 같은 시간대에 최고용량에 이름.


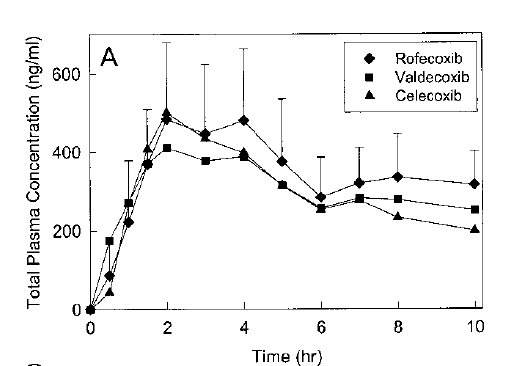

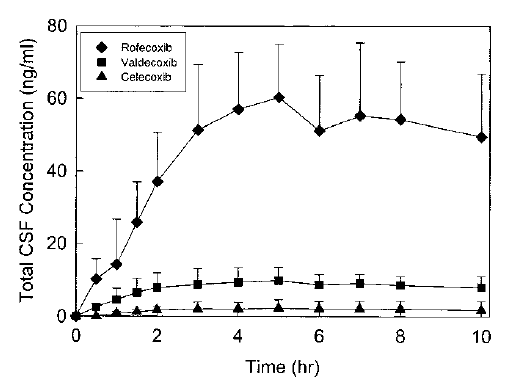


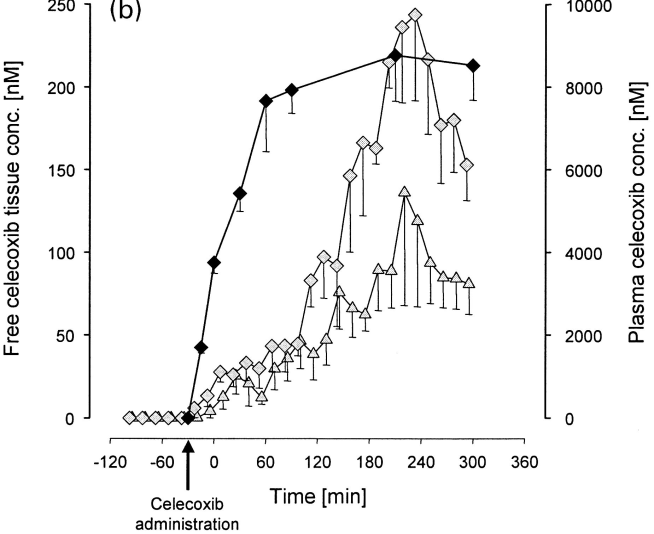


뇌조직에서 celecoxib 농도는 경구투여 후 2-4시간대에 최고치에 이르게 됨.

◎ 본 연구는 광범위하게 안전하게 쓰이는 약을 1회 복용한 전과 후, 또는 7일간 복용 전후의 뇌회로와 뇌피질의 뇌세포 전기활동 정도를 비교, 평가하는 연구이며, 임상에서 사용되는 검사도구를 이용하여 뇌의 전기생리학적인 지표의 변화를 보는 간단한 프로토콜을 사용하기 때문에, 안전하면서도 신속한 결과의 도출이 가능한 연구임.

**2) 연구 가설 및 목적**

동물모델을 대상으로 한 선행연구에서 뇌전증 발작을 줄이는 효과가 입증된 celecoxib을 건강한 성인에 투여한 후 전기생리학 검사도구를 이용하여 뇌피질의 흥분성이 감소됨을 확인하고자 함.

◎ 1차 목적

- Celecoxib 의 단회 투여에 의한 건강인의 뇌피질 흥분성 감소 효과 규명

- Celecoxib 의 1주일간의 투여에 의한 건강인의 뇌피질 흥분성 감소 효과 규명

◎ 2차 목적: Celecoxib 투여 후, 뇌의 각 부위별, 뇌파의 파장별 변화 양상 규명

1. **임상연구용 의약품 및 의료기기 코드명(또는 주성분의 일반명), 원료약품의 분량, 제형 등 (대조약 포함)**

◎ 의약품

- 제품명: Celecoxib (쎄레콕십)

- 일반명: Celebrex^®^

- 분량 및 제형: 200mg capsule

◎ 의료기기

- 뇌파:

Conventional EEG (10-20 system), (Grass-Telefactor, USA)

- 운동유발전위 (경두개자기자극기)

Magstim 200 devices (Magstim Co. Ltd., Dyfed, S. Wales, UK).

8자 코일 (type 9925, maximal output 2.2 Tesla)

1. **연구대상자의 선정 기준, 제외기준, 목표한 대상자 수 및 산출 근거**

**1) 선정기준**

1. 20세 이상 50세 이하의 남녀

2. 신체질량지수 16.0 이상 30.0 kg/m^2^ 이하

**2) 제외기준**

1. 심장질환, 뇌졸중 등 심뇌혈관 질환자

2. 간 질환자

3. 위장출혈, 염증성 장 질환자

4. 발작과 뇌전증(간질)병력

5. 최근 1주일 이내 모든 약물 복용력

6. 약물 알러지 (두드러기, 안면부종, 쇼크 등) 병력

7. 임신 중이거나 수유중인 자

8. 심전도의 임상적으로 유의미한 이상

9. AST/ALT ≥ 정상 상한치의 2배

10. 혈청 크레아티닌 ≥ 정상 상한치의 1.5배

11. 고칼륨혈증 (≥ 5.5 mmol/L)

12. 갈락토오스 불내성

**3) 목표한 대상자 수 및 산출 근거**

◎ 용량 (200mg 또는 400mg) 산출근거: 이전 연구에서 200mg 경구투여 4시간 후에, CSF농도가 2ng/ml(=5nM)이고, Ki=3-7nM 이며, 조직 내 농도가 4시간 후에 최고점에 도달하며, Ki 인 3-7nM 보다도 월등히 높으므로 200mg으로 충분한 효과를 기대할 수 있음. 하지만, 이전 연구는 서양인을 대상으로 관찰한 연구이며, CYP2C9 의 인종별 다형성을 고려해야 하고, 공복을 유지하기는 하나, 잔류 음식물에 의한 영향 또한 클 것으로 추정됨. 또한 Ki 수치는 항염증반응의 수치이며 이로 인한 전기생리학적 변화 효과는 단언하기 어려움. 그러므로, 단회 투여에 의한 뇌파 파워스펙트럼의 급성변화를 확인하기 위해서, 이보다 고용량의 투여가 필요하리라 예상함. 이전 대장암 환자를 대상으로 한 임상시험 (Bertagnolli et al) 에서 하루400mg 2회의 장기투여가, 하루 200mg 2회의 투여에 비해 이상반응의 빈도를 유의하게 증가시키지 않았으므로, 건강인에서 400mg단회투여의 위험성이 크지 않다고 추정되는 바, 본연구에서 400mg의 적용이 적절하다고 판단됨.

◎ 본 연구는 이전에 시행되었던 유사 연구가 존재하지 않으며, 본 시험약제의 뇌파, 운동유발전위의 변화 정도를 예측하기가 불가능하므로, 연구대상자 수의 산정이 어려우며, 파일럿 연구의 성격이 있음.

◎ 다른 약물을 사용하여 시행한 유사한 프로토콜 연구를 참고하면 (Joo, 2008) 평균 차이는 0.4, 각 그룹의 표준편차는 0.4, 0.2 이였음. 유의수준 0.05, 검정력 80% 로 양측 paired 또는 unpaired t test를 이용하여, Open EPi(ver3)을 통해 계산한 결과, 각 군당 10명이 적당하며, 네 개의 그룹이므로 총 40명이 필요함.

**4) 연구 대상자 모집 계획**

연구대상자는 다음과 같은 방법으로 모집한다.

◎ 서울대학교 또는, 서울대병원 내 게시판 모집공고

◎ 주요일간지, 무가지 모집공고

공고문 양식은 다음과 같다. (별첨1)

1. **연구 방법**
2. **구체적인 연구방법**

스크리닝 혈액검사를 통해 연구대상자가 연구에 적합하고, 연구참여에 동의하면 무작위 배정을 통해 다음과 같이 각 임상시험군에 배정된다. (표1)

**표1.**

| **연구1** | **연구2** |
| --- | --- |
| Celecoxib 1 군 **(10 명)** | Celecoxib 2 군 **(10 명)** |
| Placebo 1 군 **(10 명)** | Placebo 2 군 **(10 명)** |

**연구1 (아래 그림)**

◎ 무작위 배정 후, 아침공복상태로 뇌파검사를 실시한다. 검사 직 후, 시험약 400mg 또는 시험약과 같은 모양과 무게를 가진 위약을 복용한다. 4시간 후 뇌파검사를 시행한 후 귀가한다. 시험약 (또는 위약) 16개를 가지고 귀가한다.

◎ 대상자는, 다음 날부터 6일간 시험약 200mg (또는 위약)을 하루 두번씩 복용한다

◎ 7일째 아침에, 공복상태로 시험약(또는 위약) 1개를 복용한 후 4시간 후 방문하여 뇌파검사를 다시 시행한다.

**
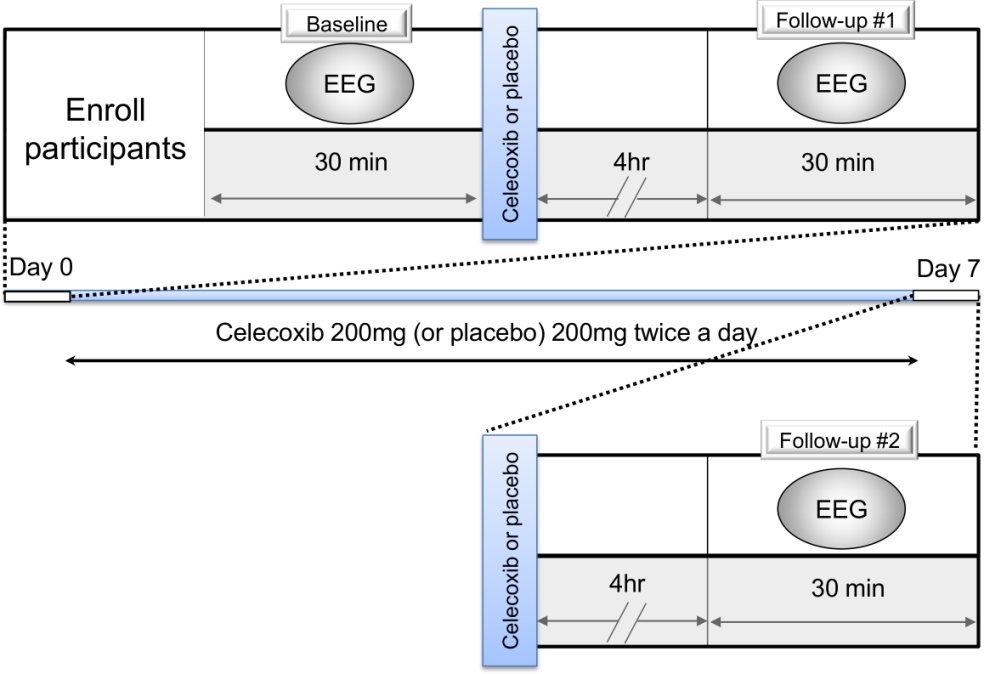
**

**연구2**

◎ 무작위 배정 후, 아침 공복상태로 오전 운동유발전위검사를 실시한다. 검사 직후, 시험 약 200mg 또는 시험약과 같은 모양과 무게를 가진 위약을 복용한다. 시험약 (또는 위약) 16개를 가지고 귀가한다. 귀가한 후 저녁에 시험약 200mg 또는 위약을 복용한다.

◎ 다음 날부터 6일간 200mg 을 하루 두번 씩 복용한다

◎ 7일째 아침에, 공복상태로 시험약(또는 위약) 1개를 복용한 후 4시간 후, 방문하여 운동유발전위검사를 다시 시행한다.

**
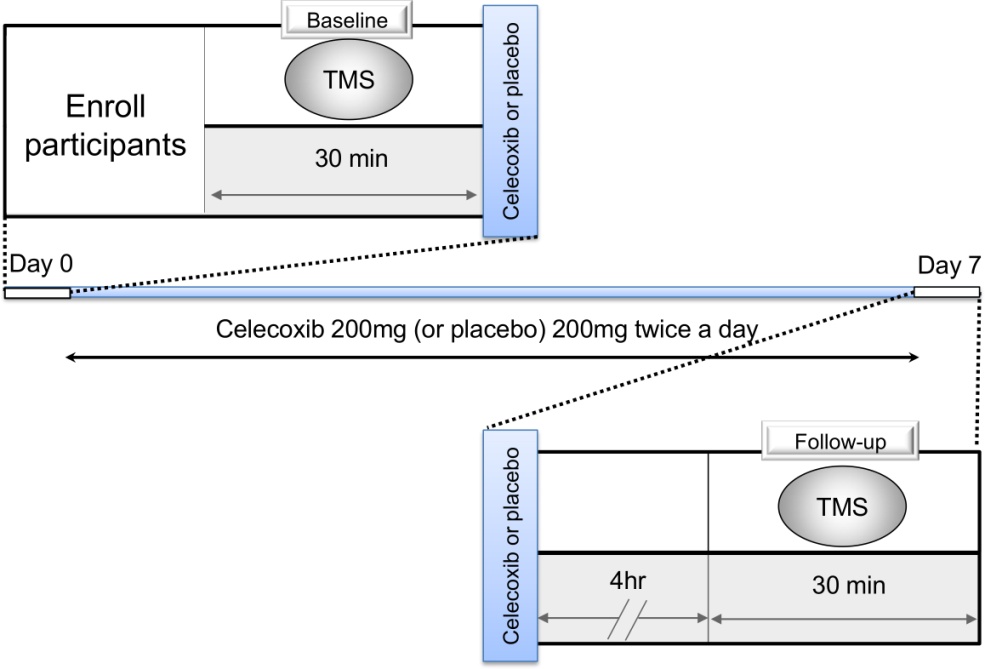
**

EEG: electroencephalography; TMS: transmagnetic stimulation

**2) 비교군 설정 및 무작위 배정 방법**

무작위배정은 이중눈가림 단계에서 사용되어 연구대상자의 시험군 배정 시 편견을 피하고, 각 시험군마다 알려지거나 미지수인 연구대상자의 특징 (즉, 인구학적 특성과 기초평가일 특성치)이 골고루 균형을 이루며, 시험군 간의 통계적 비교의 유효성을 증가시킨다. 이중눈가림 투여는 자료수집과 시험 종료 시점의 평가에서 잠재적인 편견을 줄이기 위해 사용된다. 시험 기간 동안은 연구자나 연구대상자가 연구대상자의 무작위배정 정보를 알 수 없다. 이것이 알려지면 인체적용시험 이중눈가림이 유지될 수 없기 때문이다. 무작위 배정 코드는 연구비 지원기관 (한국화이자 담당자) 에서 무작위 난수 추출 프로그램 (Research randomizer at <https://www.randomizer.org>) 을 이용하여 1부터 20까지의 난수를 생성시키고, 보관하는 것을 원칙으로 한다. (무작위배정표의 운용주체는 연구비 지원기관인 한국화이자 담당자이다.) 눈가림은 검사가 모두 종료된 시점에 해제한다. 생성된 난수를 2로 나누어 0이 남으면 celecoxib군, 1이 남으면 위약군으로 배정하고, 연구1이 종료된 뒤, 연구 2는 동일한 방법으로 시행한다. 무작위 배정코드는 원칙적으로 모든 임상시험이 끝나고, 결과분석이 종료된 후에 해제된다. 다만, 연구대상자의 투여상황을 알아야 하는 응급상황일 경우에는 이중눈가림을 해제할 수 있다. 이중눈가림을 해제하게 된 날짜, 시간, 이유를 증례기록서와 근거문서의 적절한 곳에 문서화해야 한다.

**3) 시험약 투여∙사용량, 투여∙사용 방법, 병용 요법, 대조약 사용시 그 선택사유**

연구1 : Celecoxib 또는 위약 400mg 1회, 이후 7일간 200mg, 2회/1일, 경구투여

연구2: Celecoxib 또는 위약 200mg, 2회/1일, 7일간, 경구투여

**4) 관찰항목, 임상검사항목 및 관찰검사방법**

**연구1**

◎ 관찰 항목

- 인구학적 정보: 성별, 생년월일, 연령

- 약물 투여력

- 병력

- 신체검진

◎ 임상검사항목

- 혈액 검사: AST/ALT, serum creatinine, 칼륨

- 심전도 검사

**- 뇌파검사 (electroencephalography, EEG)**

- 기존의 뇌파검사와 같은 방법으로 10-20 시스템을 이용하여 15분간 시행함

- 측정항목: 뇌파 파워스펙트럼 분석

잡음이 없고, 눈감은 각성 상태의 뇌파 중 1.5초 구간 10개를 선정하여 분석

뇌파 자료는 전두엽, 중심부, 두정엽 부위 (F, C, P)별로 분석될 것이며, 뇌파 파장밴드 별로 분석될 예정임 (Jung et al., 2011)

**일차 종점 (Primary end points)**

- 시험약 1회 단회 투여에 의한 뇌파 파워스펙트라의 변화

**이차 종점 (secondary end point)**

- 시험약 7일 투여에 의한 뇌파 파워스펙트라의 변화
- 시험약 7일 투여에 의한 뇌특정부위와 특정파장의 뇌파성질의 변화

**연구2**

◎ 관찰 항목

- 인구학적 정보: 성별, 생년월일, 연령

- 약물 투여력

- 병력

- 신체검진

◎ 임상검사항목

- 혈액 검사: AST/ALT, serum creatinine, 칼륨

- 심전도 검사

**- 운동유발전위검사 (motor evoked potential, MEP)**

- 경두개 자기자극 (Transmagnetic stimulation, TMS)을 8자 코일로 뇌운동피질 부위에 주게되며, 기록전극을 first dorsal interossei 근육에 위치시켜 파형 기록.

- 측정항목

1) Resting motor threshold (RMT): Peak-to-peak MEP 50uV 이상 나오는 가장 작은 강도 (최소4-8의 자극필요)

2) Peak to peak MEP amplitude: RMT 의 120, 140, 150% 의 강도로, 5초 간격으로 각 강도당 8번 자극하여 평균을 냄

3) 그 외 Cortical silent period (CSP), Intracortical inhibition and intracortical facilitation.

**일차 종점 (Primary end points)**

- 시험약 7일 투여에 의한 운동유발전위 항목 (resting motor potential, amplitude of MET, cortical silent period, intracortical inhibition and intracortical facilitation) 의 변화

| **연구1** | 스크리닝/  투여 전 (방문1) | 방문2(1일차) | 2일차~6일차 | 방문 3 (7일차) |
| --- | --- | --- | --- | --- |
| 동의서 | V |  |  |  |
| 인구학적 정보 | V |  |  |  |
| 약물투여력 | V |  |  |  |
| 병력 | V |  |  | V |
| 신체검진 | V |  |  |  |
| 혈액검사 | V |  |  |  |
| 심전도검사 | V |  |  |  |
| 시험약 투여 |  | V | V | V |
| 이상반응 |  | V |  | V |
| 뇌파검사 |  | V (2회) |  | V(1회) |

| **연구2** | 스크리닝/  투여 전 (visit1) | 방문2(1일차) | 2일차~6일차 | 방문 3 (7일차) |
| --- | --- | --- | --- | --- |
| 동의서 | V |  |  |  |
| 인구학적 정보 | V |  |  |  |
| 약물투여력 | V |  |  |  |
| 병력 | V |  |  | V |
| 신체검진 | V |  |  |  |
| 혈액검사 | V |  |  |  |
| 심전도검사 | V |  |  |  |
| 시험약 투여 |  | V | V | V |
| 이상반응 |  | V |  | V |
| 운동유발전위 |  | V(1회) |  | V(1회) |

**5) 효과 평가기준, 평가 방법**

◎ 분석군

- PP (per protocol)군을 대상으로 분석할 것이며, 연구계획서에 따라 “연구1” 또는 “연구2” 를 완료한 연구대상자부터의 측정된 주요변수를 분석한다.

- ITT (intention-to treat)군은 시험약 (또는 위약) 섭취 후, 최소한 1회 이상 주 평가변수에 대한 측정이 이루어진 연구대상자를 대상으로 한다.

(유효성 평가에 대한 자료는 PP군을 주 분석대상으로 하되, ITT군을 추가적으로 분석하여 유효성을 평가할 수 있다)

◎ 단회 투여 또는 7일 투여에 의한 뇌파 파워스펙트럼의 변화

- 3회 즉, 1차(baseline), 2차, 3차로 얻은 뇌파자료를 비교함. 1차와 2차의 비교는 celecoxib 의 급성 효과를 확인하게 됨. 시험약군과 위약군 투약 전후의 뇌파스펙트럼 변수의 차이값을 분석한다. 각부위별 (전두, 중심, 두정부)와 각 파장별 (알파, 델타, 쎄타, 베타파)의 차이를 분석한다.

◎ 7일투여에 의한 운동유발전위의 변화 비교

- 운동유발전위검사는 약물투여전과 7일간의 약물 투여 후에 시행하여 자료를 얻게 되며, 한 연구대상자에서 약물 복용 전후를 비교하고, 시험군과 위약군 사이의 결과를 비교한다.

**6) 기존 치료 및 연구와의 차별점**

◎ Celecoxib의 뇌신경세포 흥분억제 또는 뇌전증 발작 억제효과에 대해 논란의 여지가 있으며, 인간에서 자발발작 억제효과에 대한 연구는 전무함.

◎ 본 연구자는 in vitro, in vivo 연구를 통해, celecoxib 의 급성발작 억제를 직접적으로 증명한 바 있음.

◎ 발작은 신경세포의 자체와 신경회로 흥분성이 극도로 상승한 병적 현상으로서 celecoxib 의 발작억제 효과를 증명함이 최종 목표이나, 이에 앞서 건강인에게서 뇌피질 흥분도 및 뇌파활성의 감소를 증명하고자 함.

◎ 이미 진통소염제로서 사용중인 celecoxib의 용법, 용량을 그대로 복용하였을 때, 건강인의 뇌피질 흥분성 감소효과를 확인하는 연구는 이전 연구에서 시도된 바가 없음.

**7) 연구대상자의 이익과 위험**

◎ 투여 약물

- Celecoxib에 의해 발생되는 이상반응은 첨부한 약물설명서를 참조한다.

- 본 약제는 현재 퇴행성 관절염, 류마티스 관절염, 강직척추염을 포함하여 광범위한 급성통증에 흔히 처방되고 있는 약물임.

- FDA 승인 이전에 실시한 대규모 임상연구와 시판 후 부작용 사례를 보면, 소화불량, 얼굴부종, 복통, 구역, 발진 설사, 불면증의 부작용이 보일 수 있다고 명시되어 있음.

- 그러나 12명을 대상으로 2400mg의 투여에도 중대한 부작용이 발생하지 않음이 보고 되었음.

- 본 연구는 통증조절로 이미 승인되어 광범위하게 사용되는 용량 이내를 사용하며 특히 7일간의 비교적 짧은 기간이며, 건강한 성인을 대상으로 한다는 점에서 비가역적인 중대한 위험성이 초래될 가능성은 매우 낮다고 판단됨.

- 본 인체적용시험에서는 연구대상자의 이상반응 발생시 적절한 치료가 제공될 수 있도록 시험에 참여하는 모든 연구대상자를 대상으로 보험에 가입한다.

◎ 검사

- 뇌파검사는 발작파를 찾기 위해 흔히 시행되는 검사이며, 뇌에서 나오는 자연스런 뇌파의 기록이므로 아무런 위해가 없음.

- 운동유발전위검사는 운동신경경로의 이상을 전기생리학적으로 확인하는 검사로서, 뇌의 운동피질을 두피 위에서 자기장을 이용하여 국소적으로 자극하여 보통 손가락의 움직임을 전기적으로 기록하는 것으로 보통 척수손상 유무 또는 정도를 평가하는 검사인데, 임상적으로 안전한 자극강도가 이미 알려져 있어서 위험도가 낮은 검사임.

- 두피에 가해지는 자기자극에 의해 경미한 두통이 발생할 수 있으나 일시적이며, 짧은 검사시간을 고려할 때 부작용은 크지 않을 것으로 예상됨.

**8) 중지∙탈락 기준**

연구 기간 중, 방문 전 최소 1일 이내에, 음주를 하시거나 카페인이 들어간 음료(커피, 녹차, 에너지 드링크류)를 마신 경우, 시험대상자에게 중대한 이상 반응을 포함한 신체 이상증상이 발생한 경우, 투여 전 검사에서 발견치 못한 전신 질환이 발견된 시험대상자, 시험대상자 또는 시험대상자의 법정 대리인이 시험중단을 요구하는 경우, 복약 순응도가 총 80% 이하인 경우, 연구자나 시험대상자에 의해 중대한 시험계획(계획된 일정, 정해진 검사 등) 을 위반하는 경우, 시험대상자에게 시험제품을 투여하는데 문제가 있는 경우, 투여기간 동안 연구책임자의 지시 없이 연구 결과 판정에 영향을 줄 수 있는 약물을 복용한 경우 운동유발전위검사 중에 두통으로 인해 검사의 진행이 어려운 경우에는 임상시험을 중단하며, 시험자는 해당 시험대상자를 탈락시킨다

**9) 부작용을 포함한 안전성의 평가기준, 평가 방법 및 보고 방법**

**◎ 이상반응의 정의 (Adverse events, AEs)**

이상반응은 인체시험용 의약품/제품을 투여 받은 연구대상자에게 일어나는 모든 원하지 않은 의학적 사건이다. 이상반응은 반드시 시험제품과 인과관계를 필요로 하지는 않는다. 따라서 이상반응은 시험제품과의 관련여부와 상관없이 시험제품의 섭취와 일시적으로 관련된 바람직하지 못하고 의도되지 않은 모든 징후(비정상적인 결과 포함), 증상 또는 질환이 될 수 있다. 여기에는 새로 발생하거나 또는 기초평가일 때의 상태와 비교하여 발현 정도 또는 발현 횟수가 악화되는 것, 실험실적 검사이상을 포함한 비정상적 진단결과의 관찰도 포함된다.

**◎ 중대한 이상반응 (Serious AEs)**

ICH (International Conference on Harmonization)에 의해 내려진 중대한 이상반응의 정의는 다음과 같다:

- 사망을 초래하거나 생명을 위협하는 경우

- 입원 또는 입원 기간의 연장이 필요한 경우

- 지속적 또는 의미 있는 불구나 기능 저하를 초래하는 경우

- 선천적 기형 또는 이상을 초래하는 경우

위에 열거된 상황 이외의 경우에도 신속한 보고가 필요한지 여부를 판단하기 위해 의학적∙과학적 판단을 해야 한다. 예를 들어, 즉각적으로 연구대상자의 생명을 위협하지 않으며 사망 또는 입원을 유발하지는 않으나, 연구대상자의 상태를 악화시키고 또는 위의 정의에서 명시된 상황을 예방하기 위한 개입이 요구될 수 있다. 어떠한 이상반응이 상당히 유의한 임상적 영향을 갖는 임상징후나 증상과 연관되는 것으로 연구책임자에 의해 판단되는 경우 중대한 이상반응으로 여겨진다.

**◎** 이상반응의 중증도 평가

| 1 = 경증  Mild | 연구대상자가 거의 느끼지 못할 정도로 정상적인 일상생활 (기능)을 저해하지 않는 정도. 대부분 치료가 필요하지 않는 정도 |
| --- | --- |
| 2 = 중등도  Moderate | 연구대상자가 불편감을 느낄 수 있으며, 정상적인 일상생활 (기능)을 저해하는 정도. 연구대상자가 시험을 계속할 수는 있으나 치료가 필요할 수도 있는 정도 |
| 3 = 중증  Severe | 연구대상자가 매우 불편하여 일상생활 (기능)이 불가능하고, 시험의 계속적인 참여가 불가능한 정도. 치료나 입원이 필요할 수 있는 정도 |

**◎** 시험제품과의 인과관계 평가

| 인과관계 | 판단근거 |
| --- | --- |
| 관련 없음  (Not related) | 시험제품 섭취와 관련되지 않은 이상반응  -연구대상자가 시험제품을 섭취하지 않은 경우  -시험제품 섭취와 이상반응 발현간의 시간적 순서가 타당하지 않은 경우  -이상반응에 대해 다른 명백한 원인이 있는 경우 |
| 확실치 않음  (Doubtful) | 이상반응에 대한 다른 대체설명이 더 가능성이 큰 경우, 예를 들어 병용약물, 동반질환 또는 시간적으로 볼 때 인과관계의 가능성이 적은 이상반응  -이상반응에 대해 보다 가능성 있는 원인이 있는 경우  -섭취중단 결과(실시한 경우)가 음성이거나 모호한 경우  -재섭취결과(실시한 경우)가 음성이거나 모호한 경우 관련이 있을 |
| 가능성이 있음  (Possible) | 시험제품 섭취가 원인이 될 수 있는 이상반응. 예를 들어 병용약물, 동반질환에 의한 것인지 확실하지 않음. 시간적으로도 타당성이 있으며, 따라서 인과관계를 배제할 수 없는 경우.  -시험제품을 섭취하였다는 증거가 있는 경우  -시험제품 섭취와 이상반응 발현간의 시간적 순서가 타당한 경우  -이상반응이 다른 가능성 있는 원인들과 같은 수준으로 시험제품에서 기인한다고 판단되는 경우  -섭취중단(실시한 경우)으로 이상반응이 소실된 경우 |
| 가능성이 높음  (Probably) | 시험제품 섭취가 원인이 될 수 있는 이상반응. 시간적 연관성도 설득력이 있음(섭취 중단으로 확인됨). 예를 들어 병용약물, 동반질환에 의한 가능성이 적은 경우.  -시험제품을 섭취하였다는 증거가 있는 경우  -시험제품 섭취와 이상반응 발현간의 시간적 순서가 타당한 경우  -이상반응이 다른 원인보다 시험제품 섭취에 의해 더욱 개연성있게 설명되는경우  -섭취중단(실시한 경우)으로 이상반응이 소실된 경우 |
| 매우 가능성이 높음  (Very likely) | 시험제품의 기여 가능성이 있는 이상반응으로 명시된 이상반응으로, 다른 대체 설명에 의해서 합리적으로 설명될 수 없는 경우로서, 시간에 따른 관련성이 매 설득력이 있는 경우 (섭취 중단과 재섭취로 확인됨).  -시험제품을 섭취하였다는 증거가 있는 경우  -시험제품 섭취와 이상반응 발현간의 시간적 순서가 타당한 경우  -이상반응이 다른 어떤 이유보다 시험제품 섭취에 의해 가장 개연성있게 설명되는 경우  -섭취중단(실시한 경우)으로 이상반응이 소실된 경우  -이상반응이 시험제품 또는 동일계열의 제품에 대해 이미 알려져 있는 정보와 일관된 양상을 보이는 경우 |

**◎** 이상반응과 관련하여 취해진 조치

0 = 취해진 조치 없음

1 = 시험제품의 일시적 섭취 중단

2 = 시험제품의 섭취 중단

3 = 치료약물 병용투여

4 = 비약물치료

5 = 입원/입원기간의 연장

◎ 부작용 평가방법 및 보고방법

두번 째 방문 (Visit2) 시, 연구대상자가 약물을 복용한 이후 30분동안 즉각적인 반응을 관찰하게 되며, 운동유발전위검사가 진행되는 중과 이후 30분간까지 반응을 관찰함. 연구자는 Visual analogue scale로 운동유발전위검사의 통증 정도를 평가할 것이며, 어떤 이상반응에 대해서도 증례기록지에 기록할 것이다. 세번 째 방문 시, 연구대상자는 7일간 경험하였던 모든 증상에 대해 문진을 통해 진술하고 그 내용을 연구자가 증례기록지에 기록한다. 이상반응의 보고는 첫회 투약 이후부터 마지막 복용과 검사가 끝날 때까지로 한다. ‘중대한 이상반응’은 발견하고 24시간 이 내에 연구비 지원기관 (한국화이자제약) 에 보고 될 것이다.

**10) 자료안전성 모니터링 계획(DSMP)**

본 연구에서는 10명의 연구대상자가 등록될 때마다 연구의 안정성 및 계획서와 GCP 준수 여부를 연구의 독립성과 안전성을 해치지 않도록 최대한 객관적으로 모니터링 할 예정이다. 만약 연구 진행 도중에, SAE가 발행하게 된다면 연구자들끼리 회의를 시행하여 연구의 지속여부 및 IRB에 보고여부를 결정한다. 그러나, 본 연구는 기존에 이미 널리 사용하는 약물을 통상적인 용량으로 투약하였을 때의 효과를 비교하는 연구이며, 7일간의 짧은 연구기간에 진행되므로, 연구의 중단을 고려해야 할 SAE가 발생할 가능성은 극히 적다고 판단한다.

**11) 자료 분석 및 통계 분석 방법**

**연구1**

◎ 단회 투여 또는 7일 투여에 의한 뇌파 파워스펙트럼의 변화

- 3회 즉, 1차(baseline), 2차, 3차로 얻은 뇌파자료를 비교함. 1차와 2차의 비교는 celecoxib 의 급성 효과를 확인하게 됨. 한 연구대상자 내에서 차이를 모수검정 paired t- test 또는 비모수 Wilcoxon’s signed rank test를 통해 비교하여 유의성을 판단한다. 각부위별 (전두, 중심, 두정부)와 각 파장별 (알파, 델타, 쎄타, 베타파)의 차이분석은 repeated measures analysis of variance (rmANOVA) 을 이용한다. 연구대상자간 비교는 시험약군과 위약군간의 차이를 비교하며 student t -test 를 이용한다.

- SPSS 를 이용하여 분석될 예정이며, 유의성은 p<0.05 수준으로 판정함.

**연구2**

◎ 7일투여에 의한 운동유발전위의 변화 비교

- 운동유발전위검사는 약물투여전과 7일간의 약물 투여 후에 시행하여 자료를 얻게 되며, 한 연구대상자에서 약물 복용 전후를 비교하고, 시험군과 위약군 사이의 결과를 비교한다. paired-t test 또는 Wilcoxon's signed rank test 를 이용하고 시험군-위약군의 비교는 RmANOVA 를 이용한다.

**두 연구의 보완적 해석**

뇌파는 뇌신경세포의 시냅스 후 전위의 합으로 나타나며, 파워스펙트럼은 뇌파의 진폭과 파장이 동시에 반영되는 수치가 된다. 인체의 상태에 따라 민감하게 변동되는 수치로 작은 뇌흥분성 변화를 관찰하는데 적절한 분석방법이다. 하지만, 뇌의 각 부위별로 정상적으로 나타나는 진폭과 파장영역대가 다르기 때문에, 약물에 의한 변화가 부위별로 다르게 나타날 경우, 약물에 의한 신경억제성 효과의 해석에 상당한 제한점이 있게 된다. 운동유발전위는 운동영역에 국한된 신경세포의 기저상태 흥분성을 반영하게 되므로, 약물에 의한 뇌신경의 영향을 보다 직접적이고 명료하게 해석 가능하다.

1. **연구수행일정표**

|  | 5월이전 | 5월 | 6월 | 7 월 | 8 월 | 9 월 | 10 월 | 11 월 | 12 월 |
| --- | --- | --- | --- | --- | --- | --- | --- | --- | --- |
| - IRB승인 | V |  |  |  |  |  |  |  |  |
| - 식약처 승인 | V |  |  |  |  |  |  |  |  |
| - 연구대상자모집 및 스크리닝 |  | V | V | V | V | V | V |  |  |
| - 연구시행 |  |  | V | V | V | V | V | V |  |
| - 자료 분석 |  |  |  |  |  |  |  | V | V |
| - 결과 보고 |  |  |  |  |  |  |  |  | V |

**12. 연구대상자의 안전보호를 위한 대책**

1. **연구의 윤리성 확보를 위한 기본 방안**

◎ 시험책임자

- 본 임상시험은 시작에서부터 종료될 때까지 시험책임자 또는 시험담당자가 함께 임상시험을 적정하고 안전하게 실시될 수 있도록 만전을 기할 것이다.

- 시험책임자는 임상시험이 임상시험계획서, GCP/KGCP에 대한 현 ICH 지침 및 기타 해당 허가 요구사항에 부합하도록 보장할 책임이 있다. GCP/KGCP는 시험대상자의 임상시험 참여에 관련된 연구의 기획, 실행, 기록 및 보고에 대한 국제적 윤리 및 과학적 질의 기준이다. 이 기준의 준수는 시험대상자의 권리, 안전 및 복지에 대한 공식적인 보장, 헬싱키 선언 (2013년 개정판) 에서 비롯된 원칙의 준수, 임상시험 자료의 신뢰를 의미한다.

◎ 임상시험심사위원회 (IRB)

- 임상시험의 승인을 얻거나 승인 받은 임상시험을 변경하여 실시하고자 하는 경우, 임상시험계획서 또는 변경 계획서에 대하여 IRB의 승인을 받는다. 승인 이전에 시험대상자를 임상시험에 참여 시킬 수 없다.

◎ 이상반응 발생 시 조치

- 시험대상자의 시험 참여 도중 및 직 후, 시험책임자는 임상시험과 관련된 임상적으로 중요한 시험대상자의 이상반응에 대하여 적절한 치료가 제공되도록 해야 한다. 시험책임자는 시험대상자가 시험기간 중 의학적 조치가 필요할 때 시험대상자에게 이를 알려야 한다.

- 이상반응 발생 시에는 즉시 필요한 검사 및 치료를 받을 수 있도록 관리한다. 중대한 이상반응 발생 시에는 임상시험을 중지하고 이상반응 보고방법 및 응급절차에 따라 신속하고 적절한 조치를 취한다.

1. **연구대상자의 동의 과정**

◎ 연구책임자 또는 연구담당자, 또는 연구원이 연구대상자에게 설명하고 동의를 취득한다.

◎ 동의는 연구대상자가 직접 제공한다

◎ 연구에 대한 설명을 하고 동의를 취득하는 사이의 대기시간은 1시간 이상으로 함으로서 연구참여에 대해 충분하게 고려할 시간을 제공한다.

◎ 연구설명과정과 동의 취득과정에서 연구자는 한국어를 사용한다.

◎ 연구 대상자에게 제공되는 정보와 동의서는 다음과 같다. (별첨2)

1. **연구대상자의 보상 방안**

스크리닝 (Visit 1)을 마친 결과, 선정/배제기준에 합당하면서, 연구참여에 동의한 연구대상자는, 이후 연구를 위한 2번의 방문당 소정의 교통비 (스크리닝 방문을 제외한 방문 1회당 7만5천원, 총 15만원) 가 연구참여 종료 후에 지급받을 것이다.

1. **연구대상자의 개인정보보호 방안**

본 임상시험에서 얻어진 자료는 보고서 제출과 출판의 경우를 제외하고는 연구자가 비밀로 유지한다. 연구자는 IRB나 다른 공공기관에서 증례기록서 기록을 검증하기 위하여 연구문서를 검토하거나 복사할 수 있음을 인정한다. 또한 연구대상자는 동의서에 서명함으로써 위의 과정에 동의한다. 증례기록서 내에는 연구대상자의 이름이 아닌 이니셜 또는 배정 번호로 표기한다. 전체 데이터에 대한 접근 권한은 일차적으로 “박경일, 이상건, 정기영, 주건, 전진선”에게 주어진다. 공동연구자들은 사전에 이들에게 신고하여 허락된 경우 데이터에 접근할 수 있으나, 개인 식별이 가능한 내용은 모두 삭제된 형태의 데이터만을 열람할 수 있게 한다. 관련 기록은 연구가 종료된 시점부터 3년간 보관할 것이며, 보관기관이 지난 문서 중 개인정보에 관한 사항은 개인정보보호법 시행령 제16조에 따라 파기할 것이다.

1. **취약한 연구대상자를 포함하는 경우 추가적인 보호조치 방안**

소속기관의 연구자 직속 또는 소속기관의 피고용자가 연구에 등록될 경우 다음 사항을 준수할 것이다

◎ 연구대상자 모집의 목적으로 연구자가 피고용자와 직접적인 상호작용을 하지 않는다.

◎ 가능하다면 연구 모집이나 동의 과정은 피고용자의 상급자가 없는 상태에서 진행한다.

◎ 피고용자가 연구에 참여하지 않기로 결정할 수 있으며, 그러한 결정이 그들의 고용이나 직무 평가에 영향을 미치지 않을 것임을 알린다.

◎ 피고용자가 부당한 영향이나 강제를 받지 않을 것이며, 피고용자의 사생활이 존중될 것임을 보장할 수 있는 절차를 마련한다.

◎ 연구 참여를 거절했다는 사실이 피고용자의 상급자에게 노출되지 않도록 조치를 취해야 한다.

◎ 가능하다면 연구 진행은 다른 피고용자가 없는 상태에서 진행 되어야 한다. 예를 들어, 조사나 설문 연구에서 모든 피고용자를 한 방에 모아 놓고 현장에서 답을 하도록 요구함으로써 상급자나 동료들이 피고용자를 연구대상자로 인식하도록 만드는 방식 대신, 피고용자가 집에서 자료 작성을 완료하여 연구자에게 메일로 회신하는 방식으로 수행될 수 있다.

◎ 의뢰자의 피고용자를 연구대상자로 모집하는 경우, 의뢰자에게 제공될 모든 자료는 요약 또는 모든 개인식별 정보를 제거함으로써 피고용자의 신원이 보호되도록 해야 한다.

**13. 인체유래물의 보관 및 폐기 방법**

해당 없음

**14. 참고 문헌**

Bertagnolli MM, Eagle CJ, Zauber AG, Redston M, Solomon SD, Kim K, Tang J, Rosenstein RB, Wittes J, Corle D, Hess TM, Woloj GM, Boisserie F, Anderson WF, Viner JL, Bagheri D, Burn J, Chung DC, Dewar T, Foley TR, Hoffman N, Macrae F, Pruitt RE, Saltzman JR, Salzberg B, Sylwestrowicz T, Gordon GB, Hawk ET; APC Study Investigators. Celecoxib for the prevention of sporadic colorectal adenomas. N Engl J Med. 2006 Aug 31;355(9):873-84.

Dembo G, Park SB, Kharasch ED. Central nervous system concentrations of cyclooxygenase-2 inhibitors in humans. Anesthesiology. 2005 Feb;102(2):409-15.

Oliveira MS, Furian AF, Royes LF, Fighera MR, Fiorenza NG, Castelli M, Machado P, Bohrer D, Veiga M, Ferreira J, Cavalheiro EA, Mello CF. Cyclooxygenase-2/PGE2 pathway facilitates pentylenetetrazol-induced seizures. Epilepsy Res. 2008;79(1):14-21.

Jung KH, Chu K, Lee ST, Kim J, Sinn DI, Kim JM, Park DK, Lee JJ, Kim SU, Kim M, Lee SK, Roh JK. Cyclooxygenase-2 inhibitor, celecoxib, inhibits the altered hippocampal neurogenesis with attenuation of spontaneous recurrent seizures following pilocarpine-induced status epilepticus. Neurobiol Dis. 2006;23(2):237-46

Joo EY, Kim SH, Seo DW, Hong SB. Zonisamide decreases cortical excitability in patients with idiopathic generalized epilepsy. Clin Neurophysiol. 2008;119(6):1385-92.

Park KI, Dzhala V, Saponjian Y, Staley KJ. What elements of the inflammatory system are necessary for epileptogenesis in vitro? eNeuro March 3, 2015,

Park KI, Saponjian Y, Dzhala V, Mail M, Staley KJ. Anticonvulsant action of the cyclooxygenase inhibitor, Celecoxib in an in vitro chronic post-traumatic epilepsy model. Poster presentation at Society for Neuroscience meeting 2013.

Buysse DJ, Germain A, Hall ML, et al. EEG spectral analysis in primary insomnia: NREM period effects and sex differences. Sleep 2008;31:1673–82.

Perlis ML, Merica H, Smith MT, Giles DE. Beta EEG activity and insomnia. Sleep Med Rev 2001;5:363–74.

Jung KY, Koo YS, Kim BJ, Ko D, Lee GT, Kim KH, Im CH. Electrophysiologic disturbances during daytime in patients with restless legs syndrome: further evidence of cognitive dysfunction? Sleep Med. 2011;12(4):416-21.

Tegeder I, Niederberger E, Vetter G, Bräutigam L, Geisslinger G. Effects of selective COX-1 and -2 inhibition on formalin-evoked nociceptive behaviour and prostaglandin E(2) release in the spinal cord. J Neurochem. 2001 Nov;79(4):777-86.

Reyners AK, de Munck L, Erdkamp FL, Smit WM, Hoekman K, Lalisang RI, de Graaf H, Wymenga AN, Polee M, Hollema H, van Vugt MA, Schaapveld M, Willemse PH; DoCaCel Study Group. A randomized phase II study investigating the addition of the specific COX-2 inhibitor celecoxib to docetaxel plus carboplatin as first-line chemotherapy for stage IC to IV epithelial ovarian cancer, Fallopian tube or primary peritoneal carcinomas: the DoCaCel study. Ann Oncol. 2012 Nov;23(11):2896-902.
